# Structure-Inspired Lineage-Specific Matrix for Endogenous Neurogenesis in Spinal Cord Injury

**DOI:** 10.34133/research.0821

**Published:** 2025-08-07

**Authors:** Bo Wu, Xuejiao Lei, Xufang Ru, Jiangling Zhou, Hao Liu, Yibo Gan, Yan Wang, Wenyan Li

**Affiliations:** ^1^ School of Medicine, Nankai University, Tianjin 300071, China.; ^2^ Department of Orthopedics, General Hospital of Chinese People’s Liberation Army, Beijing 100853, China.; ^3^ Department of Orthopaedics, Xinqiao Hospital, Army Military Medical University, Chongqing, China.; ^4^ Department of Neurosurgery, Southwest Hospital, Third Military Medical University (Army Medical University), Chongqing 400038, China.; ^5^ Department of Orthopaedics, Southwest Hospital, Army Medical University (Third Military Medical University), Chongqing 400038, China.; ^6^ Department of Spine Surgery, Center of Orthopedics, State Key Laboratory of Trauma and Chemical Poisoning, Daping Hospital, Army Medical University, Chongqing 400042, China.

## Abstract

Spinal cord injury (SCI) poses substantial challenges, often leading to permanent disability and requiring adequate neuronal regeneration for functional repair. Decellularized spinal cord (DSC) matrices hold promise due to their native 3-dimensional (3D) structure and extracellular matrix (ECM)-derived biochemical components. However, their limited mechanical properties and insufficient availability of growth factors hinder their effectiveness. To address these limitations, this study introduces a core–shell design that reinforces DSC with a hydrogel-based matrix capable of delivering essential growth factors while preserving its natural structure. By leveraging 3D printing and electrostatic adsorption, the engineered matrix retains the topological features of DSC while introducing new topographical and neurogenic cues. These instructive cues facilitated an 11-fold increase in the number of newly generated neuronal cells, demonstrating lineage-specific neuronal regeneration in vivo. Mechanistically, the synergistic effects of ECM-inspired structure and biochemical cues activated the ITGA2/ITGA11–ERK/AKT signaling axis and promoted M2 macrophage/microglia polarization, thereby reducing cavity and scar formation. This optimized microenvironment enhanced endogenous neurogenesis and supported functional recovery after SCI. Overall, this study developed a structure-inspired lineage-specific matrix that effectively stimulates endogenous neuronal regeneration, highlighting its potential for advancing spinal cord repair strategies.

## Introduction

Spinal cord injury (SCI) poses formidable challenges in neurology [1], often resulting in permanent sensory and motor dysfunction due to the intricate pathophysiology at the injury site [2]. The initial trauma triggers a cascade of inflammatory responses, creating a dynamic damage microenvironment that leads to neuronal damage and glial scar formation [3–6]. Although the adverse microenvironment after SCI may induce the proliferation of endogenous neural stem cells (NSCs) in the injured area, studies have shown that 95% of endogenous NSCs differentiate into glial cells and rarely differentiate into neuronal lineage [7–9]. This situation poses a substantial challenge in generating sufficient neurons in the injured area, which is essential for the establishment of functional neural relays and motor recovery [8,10,11]. Therefore, it is necessary to create a favorable microenvironment to enhance neuronal lineage regeneration for SCI repair [12].

The spinal cord extracellular matrix (ECM) plays a key role in regulating neurogenic lineage regeneration by providing biochemical and mechanical cues (e.g., stiffness, stress relaxation, and viscoelasticity) [13–15]. Its native 3D architecture supports NSC adhesion and migration via integrin clustering and cytoskeletal rearrangement [16,17], while various neurotrophic factors such as laminin and FN1 promote cell adhesion and neuronal differentiation by binding to integrins [18,19]. Notably, decellularization of the spinal cord preserves essential ECM components such as tissue-specific nanofibers and functional matrix macromolecules [13]. The resulting decellularized spinal cord (DSC) exhibited a reduction in ECM-associated negative regulatory proteins, such as chondroitin sulfate proteoglycan (CSPG), along with diminished levels of neurotrophic secreted proteins [19]. Currently, DSC is generally dissolved and integrated with neurotrophic factors to mimic the ECM environment for neural regeneration, which even improves the long-term survival and maturation of spinal cord organoids in vitro [13,15,20,21]. Notably, due to the difficulty in fully mimicking the topology of ECM, the application of dissolved DSC in vivo has failed to achieve neurogenic lineage commitment in SCI repair [18,21–23].

Based on the above concepts, improving intact DSC has emerged as promising for mimicking ECM’s neurogenic niche. The natural topological structure of intact DSC offers asymmetric cues to guide NSC movement and axon elongation [24]. However, the mechanical instability and insufficient neurotrophic factors of DSC limit its effectiveness in achieving neurogenic lineage commitment [25–27]. Unmodified DSC has a soft, fragile structure that collapses upon implantation, leading to loss of topology and reduced neuronal differentiation—from 54% in vitro [20] to negligible levels in vivo [28]. Notably, while the soft mechanical property of DSC is conducive to neuronal differentiation [17], it poses a challenge to preserve both the structural integrity and beneficial mechanical properties of the DSC in vivo [29]. Therefore, it is necessary to develop a novel design to preserve the natural structure of DSC in vivo while improving its mechanical and biochemical cues to achieve neuronal lineage regeneration following SCI.

In this study, we developed a lineage-specific matrix designed to enhance endogenous neurogenesis by replicating ECM topology and remolding the optimal bioactive post-SCI microenvironment. Comprehensive characterization confirmed its ECM-inspired topographic structure and enhanced growth factor retention. Both in vitro and in vivo experiments confirmed that the matrix activated the ITGA2/ITGA11–extracellular signal-regulated kinase (ERK)/AKT axis and improved the inflammatory microenvironment at the injured site, significantly enhancing NSC proliferation in the matrix and promoting neuronal differentiation in 46% of newly formed cells. Moreover, motor function and electrophysiological evaluations confirmed its role in restoring functional neural relays. We propose that the synergistic combination of natural and synthetic biomaterials optimizes the microenvironment for SCI repair, representing a promising advancement in neural tissue engineering.

## Results

### Fabrication and characterization of the lineage-specific matrix

**Fig. 1.**
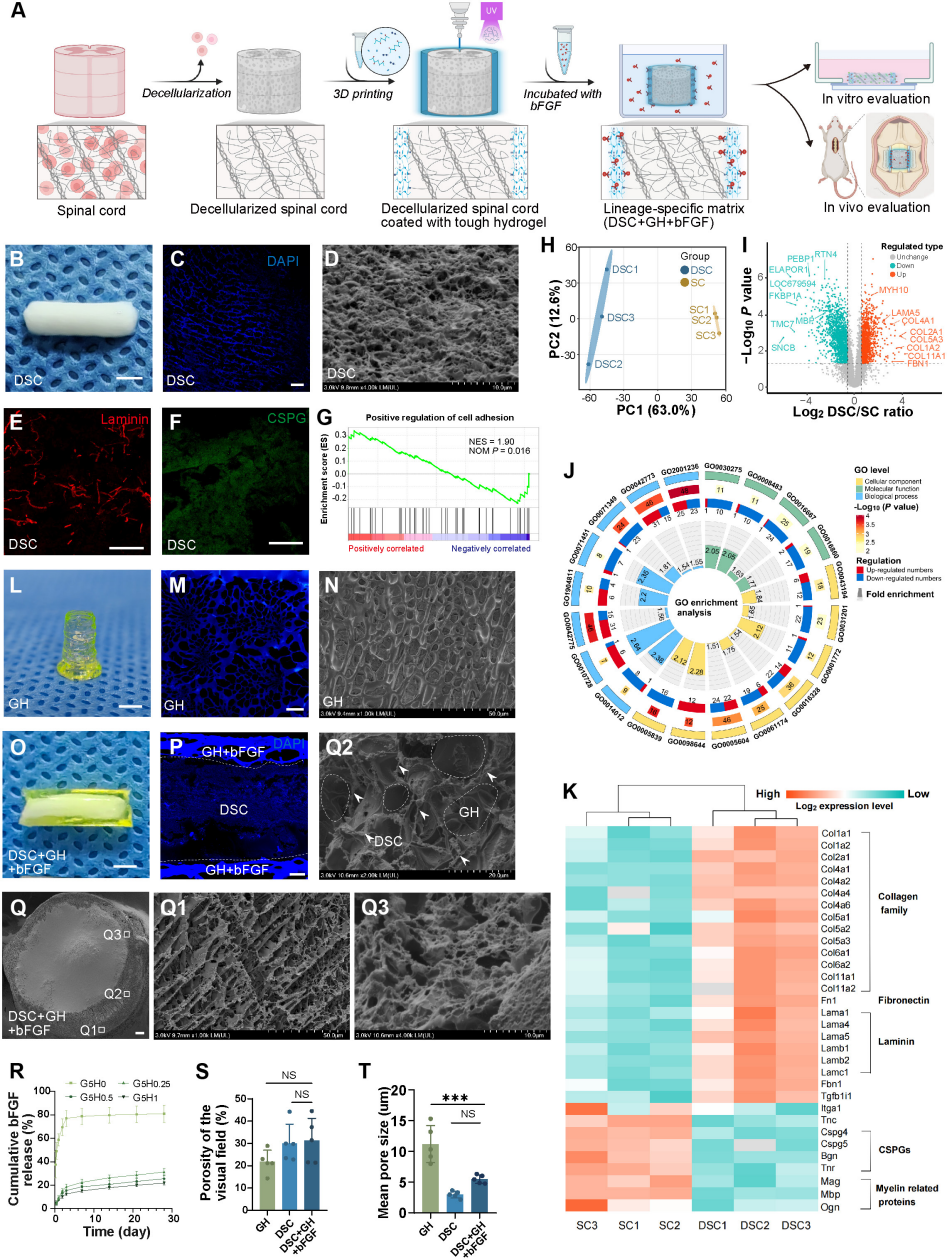
Fabrication and characterization of the lineage-specific matrix. (A) The diagram showed the construction of lineage-specific matrix. Briefly, the adult rat spinal cord was lyophilized after decellularization, wrapped with a 3D-printed composite photosensitive pregel shell, then cross-linked by UVA irradiation, and finally incubated with growth factors to obtain the lineage-specific matrix (DSC + GH + bFGF) for further research. (B and C) Photograph and DAPI fluorescence of DSC, indicating no nuclear fluorescence signal detection. (D) Cryo-SEM showed the network structure of DSC. (E) Immunostaining showed laminin retained in DSC. (F) Immunostaining showed scarce CSPG signal in DSC. (G) Proteomic GSEA analysis revealed up-regulation of proteins that promote cell adhesion in DSC. (H) PCA of proteomic data from DSC and adult spinal cord (*n* = 3). (I) The Volcano plot showed the up-regulated proteins (orange) and down-regulated proteins (green) between DSC and spinal cord (SC). (J) The functional enrichment analysis of differential proteins showed the top 20 GO pathways with the most significant difference in expression. (K) Heatmap showed the difference in the expression of ECM-related proteins between DSC and SC. (L and M) Photograph and fluorescence of GH hydrogel. (N) Cryo-SEM showed the internal structure of the GH hydrogel. (O and P) Photograph and DAPI fluorescence of DSC + GH + bFGF scaffold, wherein the dotted lines indicate the interface between DSC and hydrogel. (Q) Cryo-SEM showed the general morphology of the lineage-specific matrix, wherein Q1 showed the hydrogel shell, Q2 showed the interface between hydrogel and DSC, and Q3 showed the DSC inside the lineage-specific matrix. The area surrounded by the dotted line in Q2 is the hydrogel, while the arrow points to the DSC network. (R) Cumulative release of bFGF from the GH hydrogels. (S and T) Histogram showed the porosity and the pore size of GH hydrogel, DSC, and lineage-specific matrix. Scale bars, 2 mm (B, L, and O) and 200 μm (C, E, F, M, P, and Q). Data are expressed as the mean ± SD; *P < 0.05, **P < 0.01, ***P < 0.001, *n* = 5.

The lineage-specific matrix was constructed using a DSC core supported by a 3D-printed hydrogel shell loaded with basic fibroblast growth factor (bFGF). As illustrated in Fig. 1A, rat spinal cords were decellularized using a combination of physical and chemical methods, followed by lyophilization to create DSC scaffolds. A GelMA (gelatin-methacryloyl) + HepMA (GH) tubular hydrogel shell was then fabricated through 3D printing, tailored to match the DSC length. The lyophilized DSC scaffolds were inserted into the pregel shell, which was subsequently cross-linked with ultraviolet A (UVA) irradiation. The resulting DSC + GH scaffolds were then incubated in a bFGF solution to obtain the final lineage-specific matrix (DSC + GH + bFGF) for further research.

DSC scaffolds were initially prepared (Fig. 1B), and decellularization efficiency was assessed through 4′,6-diamidino-2-phenylindole (DAPI) immunofluorescence staining (Fig. 1C). Unlike native spinal cords (Fig. S1A1), the DSC scaffolds exhibited no nuclear fluorescence signals, revealing the complete removal of nuclear content. This observation was corroborated by scanning electron microscopy (SEM) (Fig. 1D), which further revealed the presence of a porous network structure composed of microfilaments and sheets within the decellularized scaffolds. In contrast to the native spinal cord (Fig. S1A2 and A3), immunofluorescence analysis demonstrated that while most laminin was retained (Fig. 1E), CSPG was largely depleted (Fig. 1F), indicating a favorable biochemical shift for neurogenic support.

A comparative proteomic analysis between DSC and adult spinal cord tissue revealed substantial alterations in protein composition. Principal components analysis (PCA) revealed significant differences in the protein composition between the 2 groups (Fig. 1H), while gene set enrichment analysis (GSEA) highlighted the up-regulation of proteins involved in cell adhesion in DSC (Fig. 1G). A volcano plot of differentially expressed proteins (Fig. 1I) identified a notable enrichment of various collagen proteins. Functional enrichment analysis of differentially expressed proteins (Fig. 1J) identified the top 20 significantly altered Gene Ontology (GO) pathways (Fig. S1C). Notably, the analysis also pinpointed the collagen trimer complex (GO:0098644) as the most up-regulated cellular component. Thus, certain proteins were possibly preserved during decellularization, leading to alterations in their relative abundance and concentration post-decellularization. Heatmap analysis (Fig. 1K) further revealed increased levels of ECM-related differential proteins supportive of central nervous system (CNS) regeneration, such as collagen, fibronectin, laminin, and fibrinogen 1, while CNS-inhibitory proteins like CSPG and myelin-associated proteins were reduced, aligning with the observations in Fig. 1E and F.

To enhance DSC stability and biochemical properties, a 3D-printed GH hydrogel shell was created (Fig. 1L and Fig. S1B). The internal microstructure was visualized through fluorescence staining and cryo-SEM, which confirmed a macroporous, interconnected network within the hydrogel (Fig. 1M and N). Sustained bFGF release from the composite hydrogels was quantified through enzyme-linked immunosorbent assay (ELISA) (Fig. 1R). While G_5_H_0_ hydrogels lacking HepMA exhibited minimal sustained release, those incorporating HepMA (G_5_H_0.25_, G_5_H_0.5_, G_5_H_1_) showed extended bFGF retention for up to a month. To determine the optimal GH hydrogel composition, in vitro studies assessed cell viability and proliferation across different formulations. Live/dead staining confirmed high cell compatibility (viability > 87%, Fig. S2A and B), with no significant differences in survival over 5 d (*P* > 0.05). Notably, 5-ethynyl-2′-deoxyuridine (EdU) staining at 3 d post-seeding showed that G_5_H_0.5_ + bFGF (with 0.5% HepMA) achieved the highest proliferation rate (27.77% ± 4.52%, *P* < 0.001, Fig. S2D and E). Subsequently, DSC scaffolds were coated with the optimized G_5_H_0.5_ + bFGF hydrogel, forming the final DSC + GH + bFGF construct. Cell viability assays confirmed >90% survival of C17.2 cells on this matrix after 5 d (Fig. S2A and B), demonstrating its biocompatibility and suitability for further investigation.

To enhance the mechanical stability of the DSC, we assembled it with a hydrogel shell and irradiated it with UVA, forming the structurally reinforced DSC + GH scaffold. Following co-incubation with bFGF, the final lineage-specific matrix (DSC + GH + bFGF) was successfully developed (Fig. 1O). Immunofluorescence analysis confirmed that the hydrogel, labeled with blue fluorescence, was predominantly localized around the scaffold’s periphery (Fig. 1P), with no detectable hydrogel signal in the internal DSC core. Cryo-SEM imaging further validated the core–shell structure (Fig. 1Q), showing the hydrogel layer at the periphery (Fig. 1Q1), while the junctional region revealed cross-linking interactions between DSC nanofibers (indicated by arrows) and the hydrogel (demarcated by dashed lines, Fig. 1Q2). Quantitative analysis of porosity and pore size (Fig. 1S and T) showed no significant differences between the DSC region of the lineage-specific matrix (Fig. 1Q3) and the unmodified DSC scaffold (Fig. 1D), confirming the preservation of DSC’s natural porous network—a key feature of its topological structure.

### Lineage-specific matrix enhances the neurogenic niche of DSC scaffolds

**Fig. 2.**
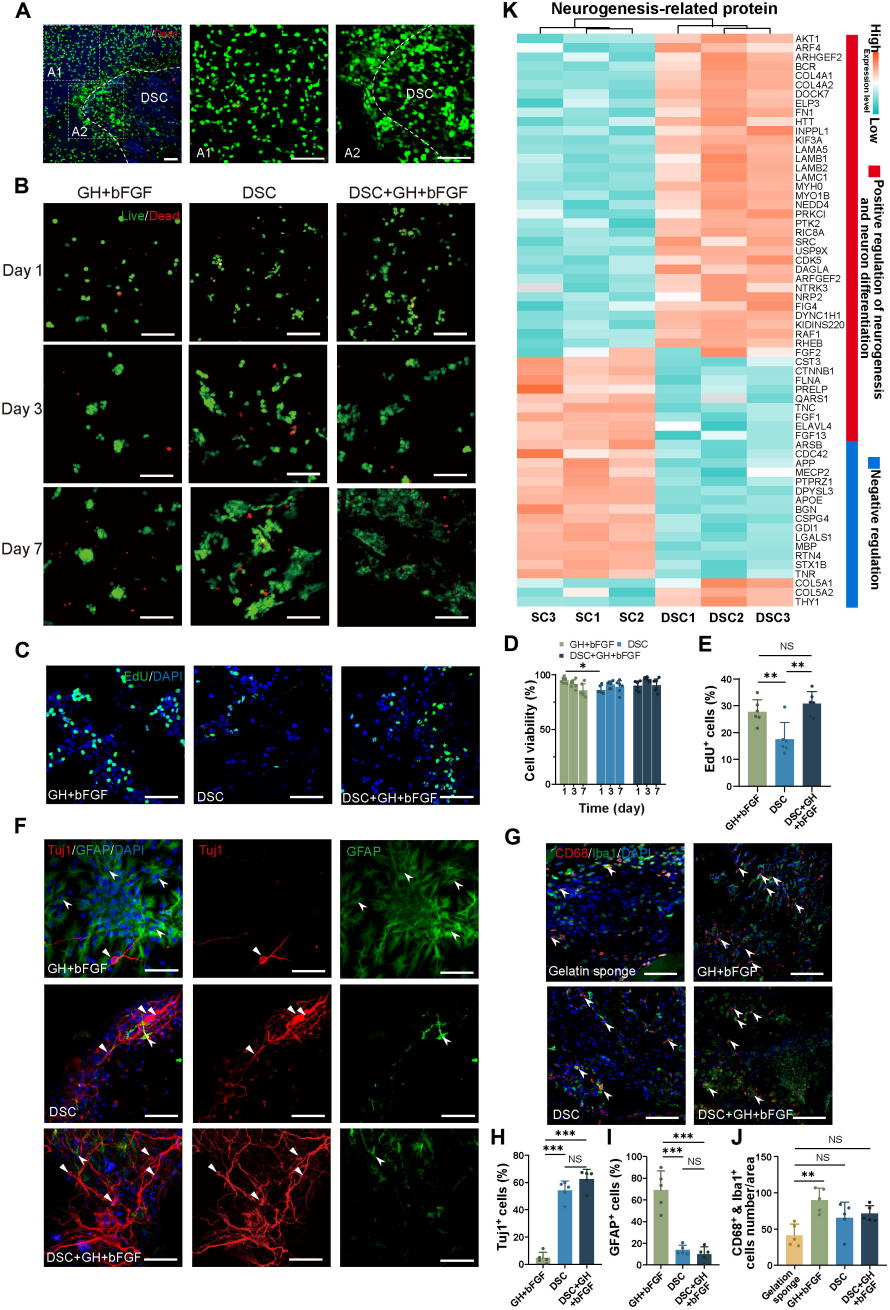
Distribution, survival, proliferation, and differentiation of cells in lineage-specific matrix in vitro and the immunogenicity of scaffolds. (A) The distribution of NSCs in a lineage-specific matrix was shown using live/dead staining, with dotted lines indicating the interface between DSC and the hydrogel. (A1) showed that cells were unevenly scattered in the hydrogel region, while (A2) showed that cells formed multilayered clusters in the DSC region. (B and D) Live/dead staining of NSCs on GH + bFGF, DSC, and lineage-specific matrix (DSC + GH + bFGF) showed no significant difference in 7-d NSC survival rates among the 3 groups (*n* = 6). (C and E) EdU staining showed proliferating cells in GH + bFGF (G_5_H_0.5_ + bFGF), DSC, and lineage-specific matrix. The histogram showed the proliferation rate of cells on the scaffolds (*n* = 6). (F) Immunofluorescence showed NSC differentiated Tuj1-positive neurons (red, triangle) and GFAP-positive astrocytes (green, arrowheads) in the scaffolds. (H and I) Histograms showed that similar to DSC, NSCs were mainly differentiated into neurons rather than astrocytes in the lineage-specific matrix, while GH + bFGF hydrogels mainly differentiated into astrocytes (*n* = 5). (G) In vivo immunogenicity evaluation of the gelatin sponge, GH + bFGF, DSC, and lineage-specific matrix. CD68 (red) and Iba1 (green) dual immunostaining showed activated mononuclear/macrophages (arrowheads) invaded in the intramuscular implantation sites. (J) Histogram showed no significant difference in CD68 + Iba1 double-positive cell counts across gelatin sponge, DSC, and lineage-specific matrix (*n* = 5). (K) Heatmap showed proteins associated with neurogenesis in adult spinal cord and DSC. Scale bars, 100 μm (A to C and G) and 50 μm (F). Data are expressed as the mean ± SD; ANOVA for (D): total: *F* = 3.645, *P* = 0.0024. Day 1: DSC + GH + bFGF versus DSC, *P* = 0.8785; DSC + GH + bFGF versus GH + bFGF, *P* = 0.5537; DSC versus GH + bFGF, *P* = 0.0321. Day 3: DSC + GH + bFGF versus DSC, *P* = 0.5945; DSC + GH + bFGF versus GH + bFGF, *P* = 0.6543; DSC versus GH + bFGF, *P* > 0.9999. Day 7: DSC + GH + bFGF versus DSC, *P* > 0.9999; DSC + GH + bFGF versus GH + bFGF, *P* = 0.7132; DSC versus GH + bFGF, *P* = 0.9333. *P < 0.05, **P < 0.01, ***P < 0.001.

To assess cytocompatibility, we cultured NSCs on the lineage-specific matrix (DSC + GH + bFGF). After 7 d, live/dead staining (Fig. 2A) revealed irregularly scattered cells within the hydrogel region (Fig. 2A1), while multilayered cellular clusters formed on the DSC scaffold region (Fig. 2A2). This “tissueoid” clustering pattern, also observed on standalone DSC scaffolds (Fig. S2C), suggests that DSC’s topological structure plays a key role in cellular organization and adhesion.

We then seeded NSCs on 3 scaffolds (GH + bFGF, DSC, DSC + GH + bFGF) for 3D culture. Live/dead staining showed over 85% cell survival in all groups within 7 d, indicating excellent cytocompatibility (Fig. 2B). Initially, the DSC group had lower survival than the GH + bFGF (86.29% ± 3.69% versus 95.03% ± 3.05%; *P* < 0.05, Fig. 2D), but by days 3 and 7, survival rates were similar across all groups (*P* > 0.05).

To further investigate NSC proliferation, we compared 3 scaffold groups (GH + bFGF, DSC, DSC + GH + bFGF) in vitro using EdU staining (Fig. 2C). Three days post-seeding, DSC alone exhibited significantly lower NSC proliferation than GH + bFGF (17.53% ± 6.16% versus 27.77% ± 4.52%; *P* < 0.01, Fig. 2E). By contrast, the lineage-specific matrix group showed significantly higher proliferation than the DSC group (30.77% ± 4.47% versus 17.53% ± 6.16%; *P* < 0.01), demonstrating that the bFGF-loaded hydrogel effectively boosts NSC expansion.

DSC further provides beneficial biochemical cues to promote neuronal differentiation of adherent NSCs. Immunofluorescence staining (Fig. 2F, H, and I) showed that NSCs on DSC predominantly differentiated into neurons (54.12% ± 7.14% for Tuj1) rather than astrocytes [13.75% ± 4.46% for glial fibrillary acidic protein (GFAP)] in the DSC group. By contrast, the GH + bFGF hydrogel favored astrocyte differentiation over neurons (4.73% ± 4.14% for Tuj1 versus 69.28% ± 17.60% for GFAP). Importantly, the lineage-specific matrix retained DSC’s pro-neurogenic effect (62.59% ± 7.12% for Tuj1 versus 10.04% ± 6.69% for GFAP), confirming that its integration with the hydrogel shell did not compromise its neurogenic potential. Further proteomic analysis of data from DSC and fresh spinal cord samples identified key proteins involved in neurogenesis and neuronal differentiation (including neural precursor cell proliferation, GO:0061351; neuroblast migration, GO:0097402; positive regulation of neuron differentiation, GO:0045666). Heatmaps revealed that DSC retained up-regulated levels of neurogenic proteins, while inhibitory factors were down-regulated compared to the adult spinal cord (Fig. 2K). These findings suggest that DSC supports NSC survival and neuronal differentiation due to its biochemical composition in the neurogenic niche, and its integration into the lineage-specific matrix enhances these neurogenic properties by improving cell proliferation.

### Lineage-specific matrix supports the 3D structure of DSC in vivo with biodegradability and low immunogenicity

The implantation of allogeneic tissue-derived scaffolds often triggers inflammatory responses [23,30,31]. Thus, evaluating the immunogenicity of DSC-based scaffolds is crucial before in vivo application. In this study, a gelatin sponge with good biocompatibility served as the control group [32]. One week after implantation, dual immunofluorescence staining (CD68 + Iba1) detected activated invasive monocytes/macrophages, with CD68 serving as a marker for the activated macrophages (Fig. 2G). The DSC and lineage-specific matrix (DSC + GH + bFGF) exhibited comparable immune responses to the gelatin sponge (*P* > 0.05), while the GH + bFGF group showed significantly higher macrophage infiltration (*P* < 0.01, Fig. 2J). This confirms that the lineage-specific matrix has low immunogenicity, making it a suitable candidate for implantation.

**Fig. 3.**
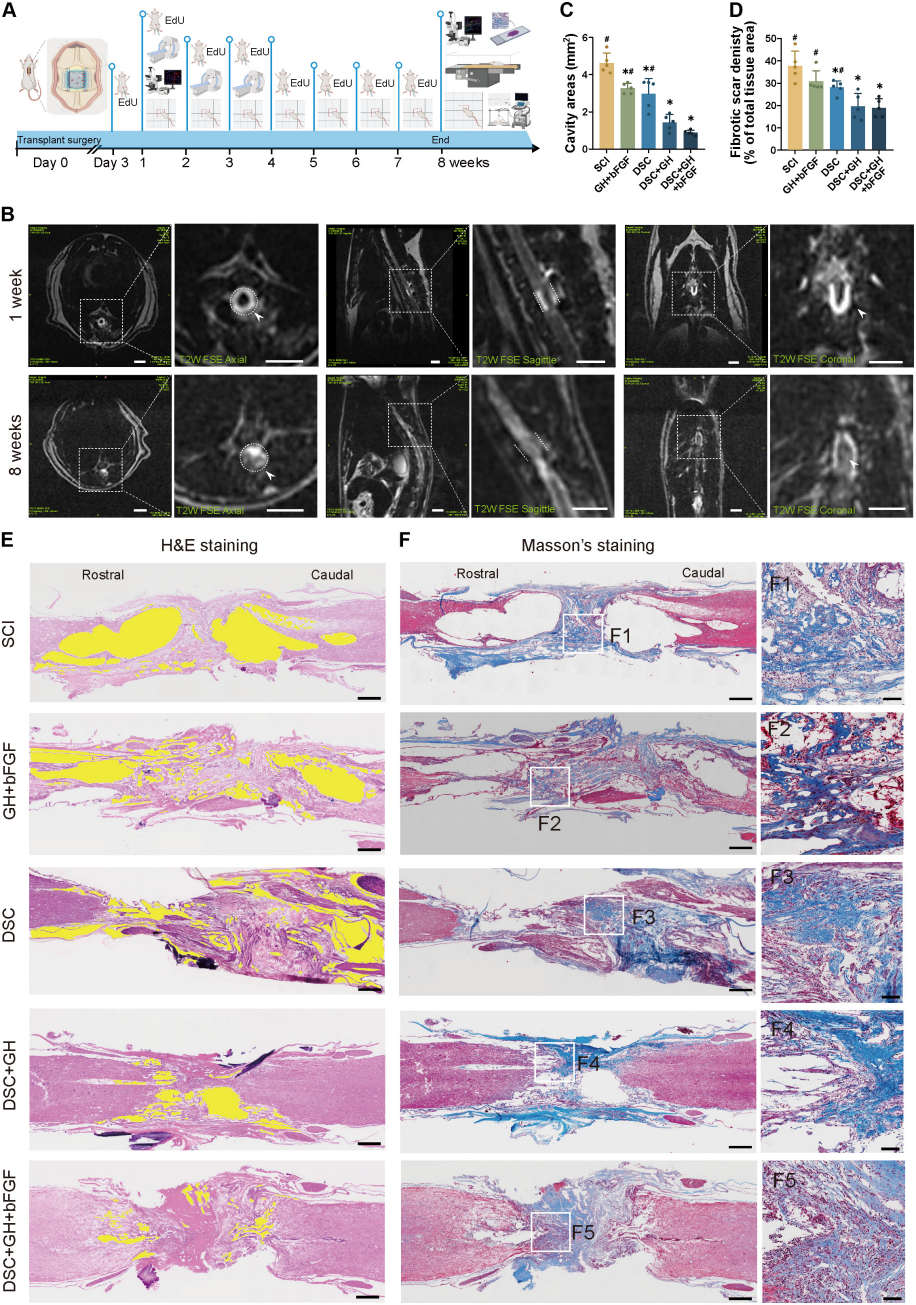
The lineage-specific matrix alleviated the formation of fibrous scars and cavities. (A) Flow chart of the lineage-specific matrix in vivo evaluation experiment. (B) Representative T2W MRI images of the spinal cord implant area at 1 and 8 weeks after lineage-specific matrix implantation, with enlarged axial, sagittal, and coronal views on the right. (C and E) H&E staining showed the cavities (filled with yellow) in the injured area 8 weeks after the implantation of the scaffolds into each group (SCI, GH + bFGF, DSC, DSC + GH, and DSC + GH + bFGF). The bar chart compares the cavity areas among these groups, showing that scaffold implantation had significantly reduced the cavity area when compared to the SCI group, with the lineage-specific matrix (DSC + GH + bFGF) producing the smallest cavities. (D and F) Masson’s staining showed fibrous scar (blue stained area) of the 5 groups (SCI, GH + bFGF, DSC, DSC + GH, and DSC + GH + bFGF), with (F1) to (F5) showing high-magnification views of the injury area. The bar chart compares the area of fibrous scar among these groups, showing that the lineage-specific matrix group had significantly reduced fibrous scar when compared to the SCI and DSC groups. Scale bars, 5 mm (B), 500 μm (E and F), and 50 μm (F1 to F5). Data are expressed as the mean ± SD; ^*^P < 0.05 versus SCI group; ^#^*P* < 0.05 versus DSC + GH + bFGF group; ^NS^*P* > 0.05; *n* = 5.

Figure 3A presents the in vivo evaluation process of the lineage-specific matrix, with experimental groups including SCI, GH + bFGF, DSC, DSC + GH, and lineage-specific matrix. Scaffolds were implanted into SCI rats, followed by weekly EdU injections to track cellular proliferation. Motor function was assessed using the Basso, Beattie, and Bresnahan (BBB) score, while magnetic resonance imaging (MRI), histological [hematoxylin and eosin (H&E), Masson’s, immunofluorescence staining], behavioral, and electrophysiological analyses were performed at time points up to 8 weeks post-injury/implantation.

The incorporation of the rigid hydrogel shell significantly improved DSC’s mechanical strength, addressing its inherent weakness in compression resistance. Movie S1 visually illustrates the improved mechanical properties across different bioscaffolds. Compared to DSC alone, the GH composite hydrogel exhibited an 11-fold increase in compression modulus (0.0240 ± 0.0071 MPa versus 0.0022 ± 0.0004 MPa; *P* < 0.001, Fig. S3A). Notably, the compressive modulus of the hydrogel shell-reinforced DSC increased significantly (0.0127 ± 0.0014 MPa versus 0.0022 ± 0.0004 MPa; *P* < 0.05). The lineage-specific matrix closely mimicked the stress–strain response of native spinal cord tissue (Fig. S3B). Its compression modulus (0.0127 ± 0.0014 MPa) was statistically similar to that of the fresh spinal cord (0.0141 ± 0.0053 MPa; *P* > 0.05, Fig. S3A), suggesting that the lineage-specific matrix successfully recapitulates the mechanical properties of native spinal tissue. Then, the lineage-specific matrices were implanted into rats, and the results showed that their compressive modulus remained relatively stable during the initial 2 weeks but significantly decreased by the fourth week (Fig. S3C and D), indicating that the matrices maintained their mechanical properties during the early phase following implantation.

Then, MRI and immunofluorescence were employed to evaluate the 3D structure and degradability of the lineage-specific matrix in vivo. One week after implantation, the hydrogel shell retained its ring-like structure, while the sagittal and coronal views showed a “sandwich-like” scaffold morphology (Fig. 3B). By the second week, MRI indicated that structural integrity was largely maintained, although gradual degradation was observed by the third week (Fig. S3E). Analysis of the outer contour of the lineage-specific matrix revealed consistent circularity between the first and second weeks, but a significant decrease in the third week, indicating that deformation began at that time (Fig. S3F). By the fourth week, the outer contour of the lineage-specific matrix exhibited significant deformation, and by the eighth week, the majority of the hydrogel shell was observed to be degraded (Fig. 3B and Fig. S3E to G). These findings were consistent with the results of immunofluorescence (Fig. S3H). Proper biodegradation minimizes chronic inflammation and aligns with the repair timeline for SCI [33,34]. Optimal SCI repair occurs before scar maturation (~14 d post-injury) [35], while fibroblast activity peaks around day 7 [36]. These findings confirm that the lineage-specific matrix provides essential structural support to DSC in the critical early stages of spinal cord repair while ensuring timely degradation to prevent long-term inflammatory complications.

### The lineage-specific matrix alleviates fibrous scar formation and cavitation

In this study, the implantation of a lineage-specific matrix effectively reduced fibrous scar formation and cavitation in the injured spinal cord. Histological evaluations using H&E and Masson’s staining at 8 weeks after surgery highlighted cavity areas in yellow pseudo-color (Fig. 3E and F). The harvested spinal cords at this time point are shown in Fig. S4A. The cavity areas and fibrous scar formation in the DSC and its derived scaffold groups were significantly smaller than those in the SCI group (*P* < 0.05, Fig. 3C and D). Compared with the DSC group, the DSC + GH group, which incorporates a hydrogel shell, further significantly reduced the cavity area and fibrous scar formation at the injury/graft site (*P* < 0.05). Notably, the lineage-specific matrix further decreased both the cavity area and fibrous scar formation compared with the DSC group (*P* < 0.05). These findings suggest that the lineage-specific matrix may facilitate endogenous neurogenesis by limiting fibrous scar invasion and reducing cavitation.

### The lineage-specific matrix promotes endogenous neurogenesis of the injury/graft area

**Fig. 4.**
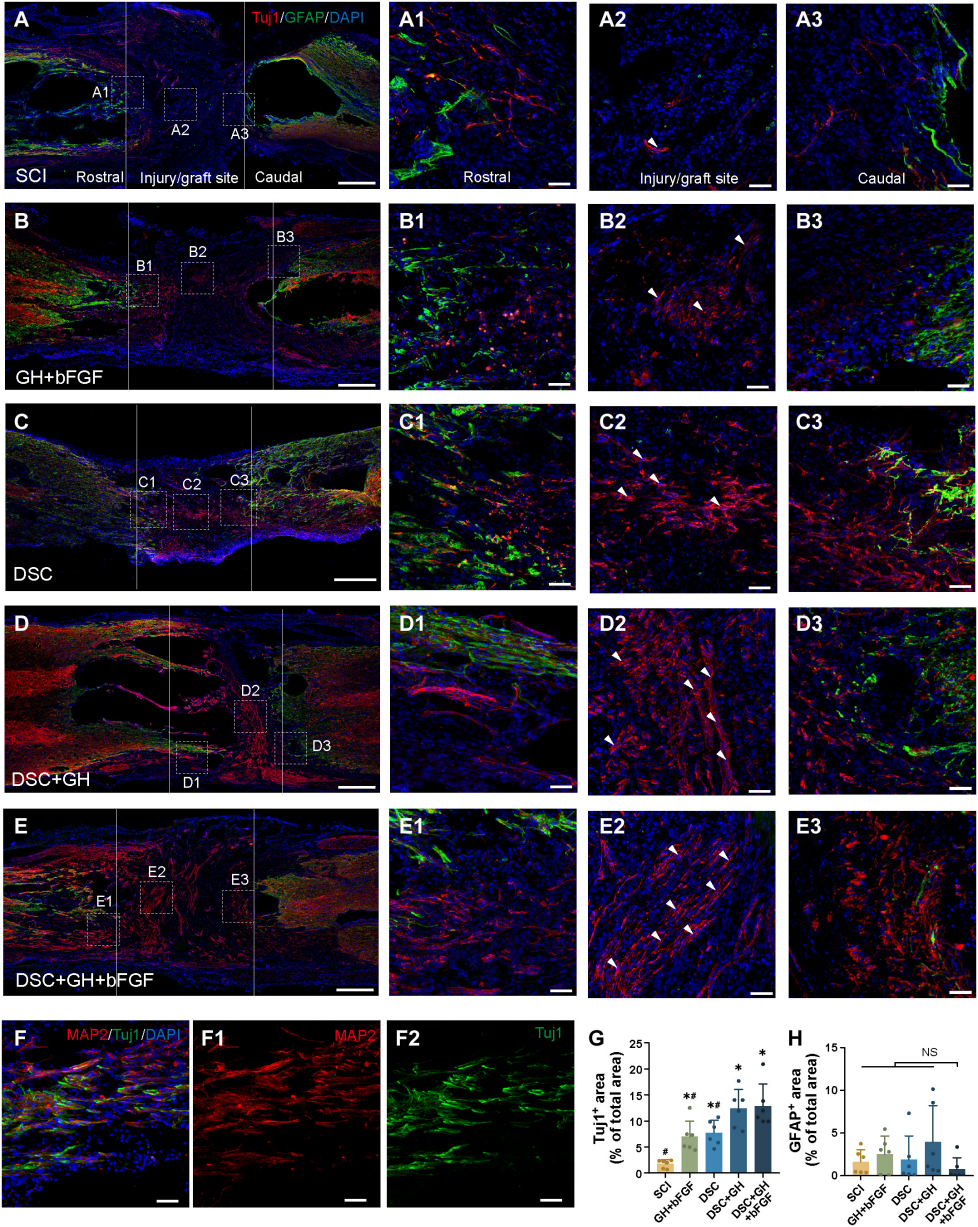
The lineage-specific matrix promoted reinnervation of the injury/graft area at 8 weeks post-implantation. (A to E) Immunofluorescence showed neurons (Tuj1, red) and astrocytes (GFAP, green) in the injury/graft area (inside the dashed lines), with Tuj1-positive neurons marked by red triangles. High-magnification views of the rostral, internal, and caudal regions are shown in (A1) to (E1), (A2) to (E2), and (A3) to (E3), respectively. Neuronal signals were observed rostrally in all groups, while abundant caudal signals appeared only in the DSC, DSC + GH, and lineage-specific matrix (DSC + GH + bFGF) groups. (F) Double immunofluorescence showed that most neurons in the injury/graft area coexpressed MAP2 (red) and Tuj1 (green). (G and H) Bar charts showed the proportion of Tuj1 or GFAP area in the injury/graft regions across 5 groups (SCI, GH + bFGF, DSC, DSC + GH, and DSC + GH + bFGF). The Tuj1-positive area was the highest in the lineage-specific matrix group, while the GFAP-positive areas showed no significant differences among the groups. Scale bars, 500 μm (A to E), 50 μm (A1 to E1, A2 to E2, A3 to E3, and F to F2). Data are expressed as the mean ± SD; ^*^P < 0.05 versus SCI group; ^#^*P* < 0.05 versus DSC + GH + bFGF group; ^NS^*P* > 0.05; *n* = 6.

At 8 weeks after injury/implantation, spinal cord sections from SCI, GH + bFGF, DSC, DSC + GH, and lineage-specific matrix (DSC + GH + bFGF) groups underwent immunofluorescence staining to identify neurons and astrocytes (Fig. 4A to F). Scaffolds in all groups significantly increased the proportion of Tuj1-positive neurons in the injured spinal cord compared to those in the SCI group (*P* < 0.05, Fig. 4A2 to E2 and G). The DSC + GH group, supported by a hydrogel shell, exhibited a significant increase in the proportion of Tuj1-positive neurons at the injury/graft site (12.40% ± 3.65% versus 7.75% ± 2.42%, *P* < 0.05). Notably, the lineage-specific matrix group exhibited the most Tuj1-positive neurons in the injury/graft area. While neuronal signals were detected rostral to the injury/graft area in all 5 groups (Fig. 4A1 to E1), abundant caudal neuronal signals were exclusively observed in the DSC, DSC + GH, and lineage-specific matrix groups (Fig. 4A3 to E3). Quantitative analysis indicated a significant increase in neuronal signals in the caudal injury/graft area for the DSC, DSC + GH, and lineage-specific matrix groups compared to the SCI group (*P* > 0.05, Fig. S4B). Double immunostaining (MAP2 + Tuj1) confirmed neuronal presence (red for MAP2, green for Tuj1). Additionally, quantitative analysis showed no significant difference in astrocyte signals at the injury center across all groups (*P* > 0.05, Fig. 4A2 to E2 and H). These results indicate that the lineage-specific matrix promotes endogenous neurogenesis in the injured spinal cord.

### The lineage-specific matrix facilitates NSC migration, neuronal differentiation, and cell proliferation after SCI

**Fig. 5.**
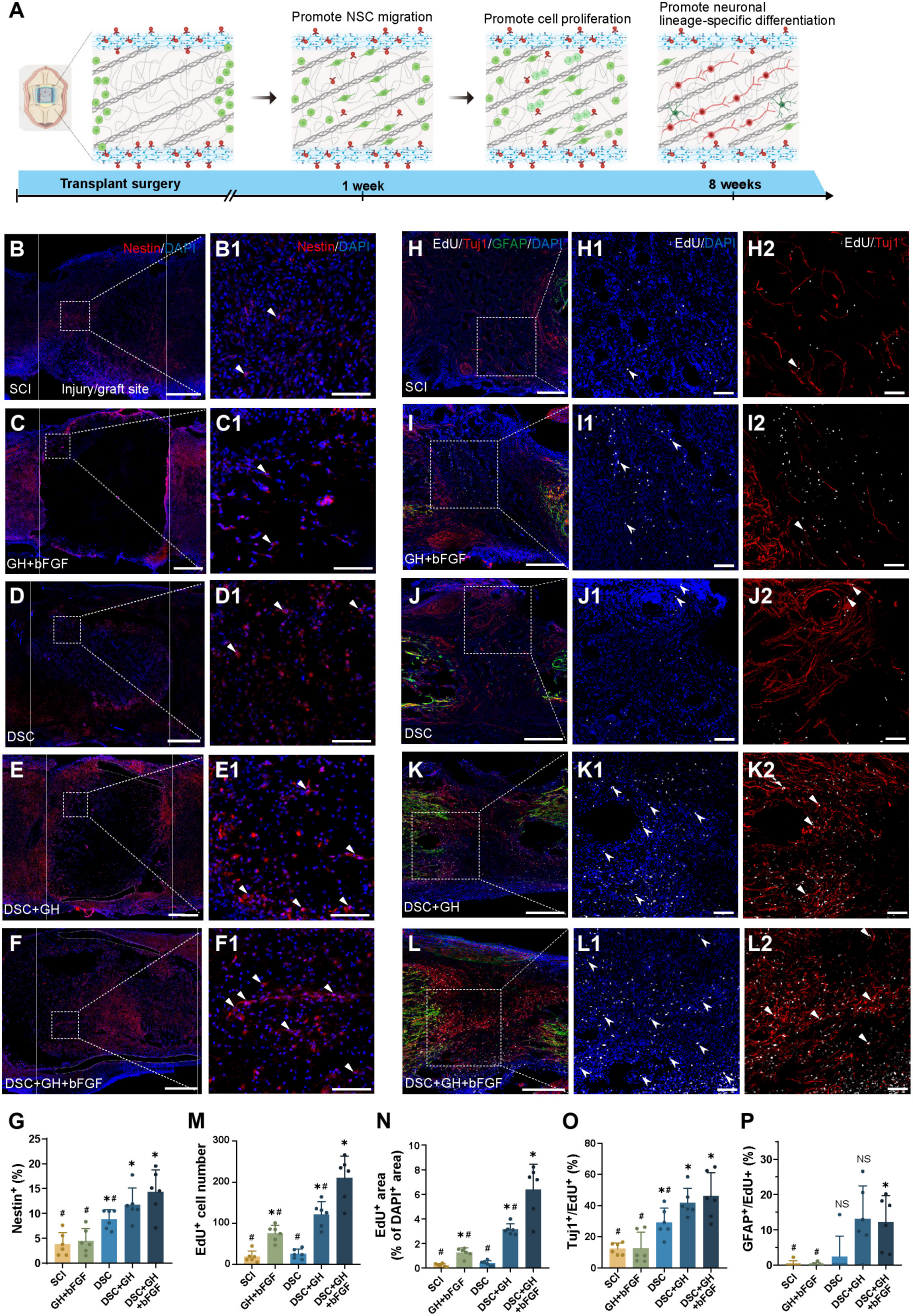
Implantation of the lineage-specific matrix facilitated the migration, neuronal differentiation, and proliferation of endogenous NSCs after SCI. (A) Schematic diagram of the reinnervation of the SCI area promoted by the lineage-specific matrix (DSC + GH + bFGF). (B to F) At 7 d after the operation, Nestin (red) immunostaining was performed in the injury/graft area of the spinal cord in each group. Dashed lines indicated the injury/graft areas, while the dashed curves outlined the hydrogel shells. (B1) to (F1) showed the high-magnification views of the boxed areas in (B) to (F), with triangles indicating Nestin-positive NSCs. (G) Bar chart showed the proportion of endogenous Nestin-positive cells in the injury/graft area of the 5 groups. (H to L) Triple immunofluorescence staining of EdU (white), Tuj1 (red), GFAP (green), and DAPI (blue) in the injury/graft area for each group 8 weeks post-injury/implantation. (H1) to (L1) showed the boxed regions in (H) to (L), indicating EdU-labeled proliferating cells (arrowheads). (H2) to (L2) showed the boxed regions in (H) to (L), indicating EdU-labeled Tuj1 neuronal cells (triangles). (M and N) Bar charts showed the number or proportion of EdU-labeled cells in the injured area of each group. The lineage-specific matrix with sustained-release bFGF was significantly better than that in both the SCI and DSC groups with respect to promoting cell proliferation. (O and P) Bar charts showed the proportion of Tuj1- and GFAP-positive cells among EdU-labeled cells in the injured area for each group. When compared with the SCI and GH + bFGF groups, DSC and the derived scaffolds promoted most of the newly proliferated cells to differentiate into neural cells. In addition, the lineage-specific matrix promoted a small portion (12.2%) of newly proliferating cells to differentiate into astrocytes. Scale bars, 500 μm (B to F and H to L) and 100 μm (B1 to F1, H1 to L1, and H2 to L2). Data are expressed as the mean ± SD; ^*^P < 0.05 versus SCI group; ^#^*P* < 0.05 versus DSC + GH + bFGF group; ^NS^*P* > 0.05; *n* = 6.

Figure 5A illustrates the proposed mechanism by which the lineage-specific matrix (DSC + GH + bFGF) promotes reinnervation in the SCI area. The matrix induces endogenous NSC migration to the injury site, while sustained-release bFGF and hydrogel shell support enhanced neuronal lineage-specific regeneration post-SCI. Immunofluorescence staining for Nestin assessed NSC migration 1 week after injury/implantation (Fig. 5B to F). The GH + bFGF group exhibited no significant change in NSC proportion compared with the SCI group (*P* > 0.05), whereas the DSC, DSC + GH, and lineage-specific matrix groups exhibited significant increases (*P* < 0.05, Fig. 5B1 to F1 and G). In addition, the lineage-specific matrix group had significantly higher NSC numbers than the SCI group (*P* < 0.05, Fig. S4C). These results indicated that the lineage-specific matrix facilitated early endogenous NSC migration to the injury site after SCI. Double staining for SOX2 and Nestin further confirmed NSC presence (Fig. S4D).

To evaluate cell proliferation in vivo, EdU was administered intraperitoneally at 3 days post-injury (dpi) and weekly thereafter. At 8 weeks after injury/implantation, triple immunofluorescence staining assessed proliferating cells (Fig. 5H to L). EdU-positive cells represented newly proliferating cells from 3 d to 8 weeks after injury (Fig. 5H1 to L1). No significant difference in proliferating cell numbers and proportions was observed between the SCI and DSC groups (*P* > 0.05, Fig. 5M and N). Interestingly, compared with the DSC group, the DSC + GH group showed significantly increased proliferation (*P* < 0.05). This finding suggests that DSC cell proliferation can be restored by preserving its topological structure within a hydrogel shell. Moreover, the lineage-specific matrix displayed optimal cell proliferation after SCI (6.39% ± 2.06% versus other groups, *P* < 0.05), indicating that topographical and biochemical cues synergistically enhanced DSC cell proliferation.

Triple immunofluorescence staining (Tuj1, GFAP, and EdU) assessed newly proliferating cell differentiation in the SCI area. The lineage-specific matrix had the highest proportion of Tuj1^+^ neurons among EdU-positive cells, followed by DSC + GH and DSC groups, and all significantly different from the SCI group (12.4% ± 3.78% versus others; *P* < 0.05, Fig. 5H2 to L2 and O). Notably, the proportion of Tuj1^+^ neurons among newly proliferating cells was significantly higher in the lineage-specific matrix group than in the DSC group (*P* < 0.05), whereas the DSC + GH group showed no significant increase (*P* > 0.05). Based on proliferating cell numbers and newborn neuron proportions, the number of newborn neurons in the lineage-specific matrix group was 12.9 times greater than in the DSC group. This suggests that optimizing DSC biochemical composition and leveraging its topological structure further enhanced neuronal lineage differentiation of migrating NSCs post-SCI. Additionally, the lineage-specific matrix modestly promoted astrocyte differentiation (Fig. S4E and Fig. 5P). Overall, this matrix provides essential cues for NSC migration, proliferation, and neuronal differentiation, thereby promoting neural regeneration.

### The lineage-specific matrix modulates macrophage/microglia activation and induces M2 polarization after SCI

**Fig. 6.**
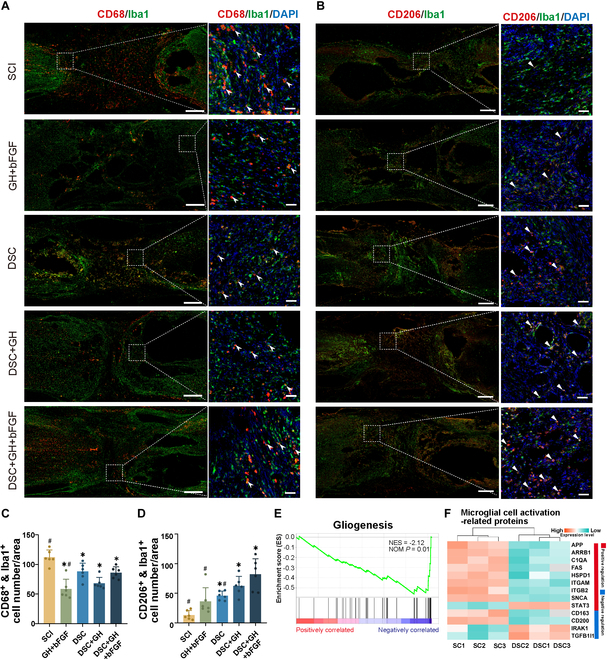
Implantation of the lineage-specific matrix regulated the activation of macrophages/microglia and induced M2 polarization at 8 weeks after SCI. (A) CD68 (red) and Iba1 (green) immunostaining highlighted activated macrophages/microglia (arrowheads) in the injury areas across groups (SCI, GH + bFGF, DSC, DSC + GH, DSC + GH + bFGF), with right-side images showing magnified views of the boxed areas. (B) CD206 (red) and Iba1 (green) immunostaining indicated M2-polarized microglia (triangles) in the injury areas across groups, with right-side images showing magnified views of boxed areas. (C) Bar chart showed that the numbers of activated microglia (CD68 + Iba1 double positive) in the injured area of the other groups were less than that of the SCI group. (D) Bar chart showed that the numbers of M2 microglia (CD206 + Iba1 double positive) in the injured areas of the DSC, DSC + GH, and lineage-specific matrix groups were higher than that of the SCI group. (E) Proteomic GSEA analysis showed that DSC was significantly down-regulated in the gliogenesis pathway when compared with that in the adult spinal cord. (F) Heatmap showed proteins associated with microglia activation in adult spinal cord and DSC. Scale bars, 500 μm (A and B, left) and 50 μm (A and B, right). Data are expressed as the mean ± SD; ^*^P < 0.05 versus SCI group; ^#^*P* < 0.05 versus DSC + GH + bFGF group; ^NS^*P* > 0.05; *n* = 6.

At 8 weeks after implantation, double immunostaining identified macrophages/microglia in the injury/graft area, with CD68 + Iba1 indicating activated microglia (Fig. 6A) and CD206 + Iba1 indicating M2-polarized microglia (Fig. 6B). The DSC, DSC + GH, and lineage-specific matrix groups exhibited fewer activated microglia and more M2 microglia in the injured area than the SCI group (*P* < 0.05, Fig. 6C and D). Although no significant differences were observed in the number of activated microglia among the DSC, DSC + GH, and lineage-specific matrix groups (*P* > 0.05, Fig. 6C), the number of M2 microglia in the injured area of the lineage-specific matrix group was significantly higher than that in the DSC group (*P* < 0.05, Fig. 6D).

The ability of the lineage-specific matrix to inhibit microglial activation and induce M2 polarization is related to the protein composition of DSC. GSEA of proteomic data revealed significant down-regulation of gliogenesis pathway in DSC compared with the adult spinal cord (Fig. 6E). A heatmap also unveiled that proteins promoting microglial activation were markedly reduced in DSC relative to the adult spinal cord (Fig. 6F). These findings suggest that the lineage-specific matrix establishes a tissue repair-conducive microenvironment by modulating microglial activation and inducing M2 polarization.

### Implantation of lineage-specific matrix improved the motor function of rats after SCI

**Fig. 7.**
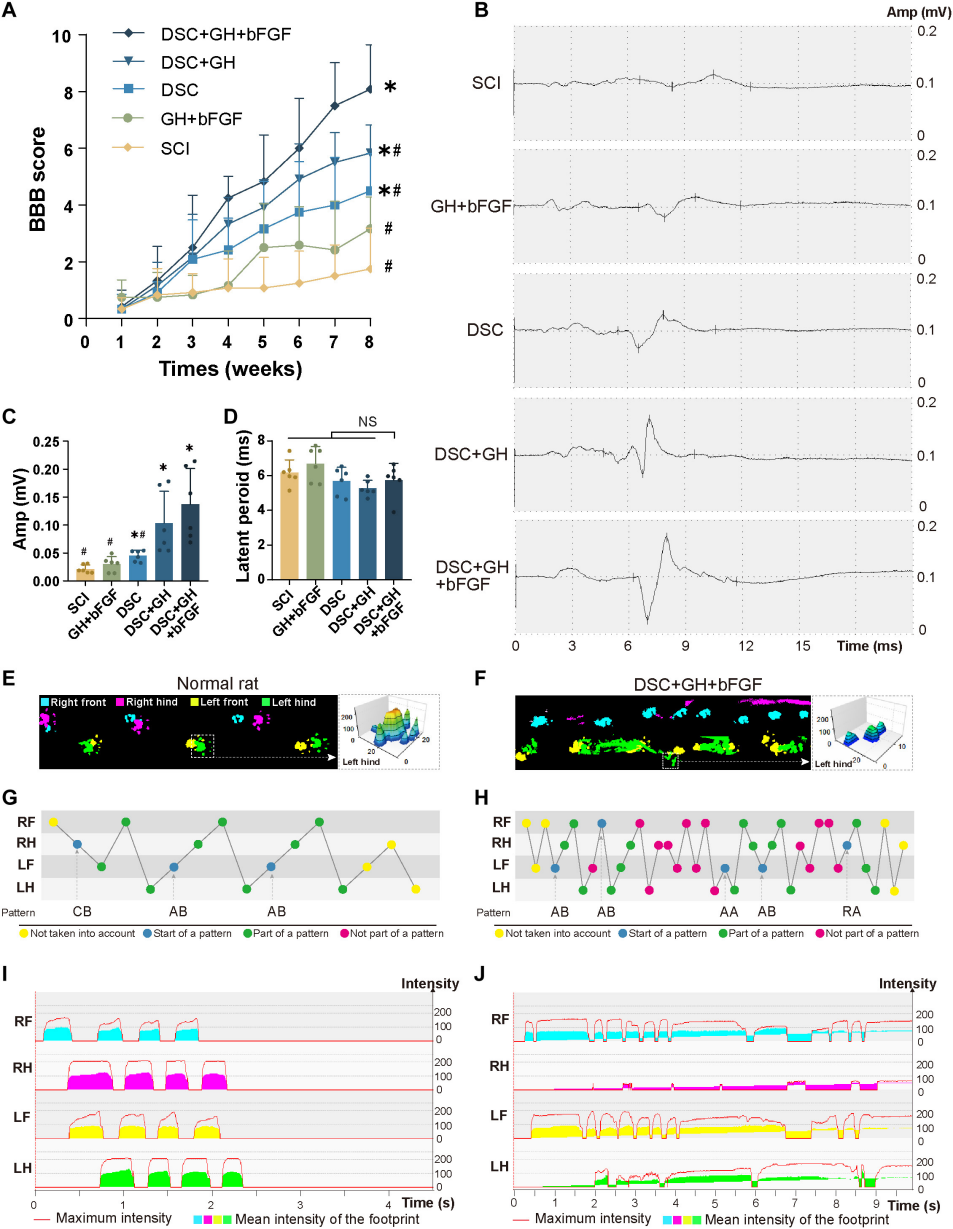
Implantation of lineage-specific matrix improved the motor function of rats after SCI. (A) BBB scores of rats weekly for 8 weeks after SCI or scaffold implantation. (B) Representative mMEP of rats in each group at 8 weeks after surgery. (C) Bar chart showed that the amplitudes of DSC, DSC + GH, and lineage-specific matrix (DSC + GH + bFGF) were significantly higher than those of the SCI group. (D) Bar chart showed that there is no significant difference in the latent period among the groups. (E) Footprint and representative 3D footprint intensities of the normal rat. (F) Footprint and representative 3D footprint intensities of the rats at 8 weeks after lineage-specific matrix implantation, showing that the hindlimb was mainly bearing on the palm of the foot and the big toe. (G and H) Step sequence analysis showed uncoordinated steps in the lineage-specific matrix group when compared to that in normal rats. (I and J) Footprint intensity curves for normal rats and those with lineage-specific matrix 8 weeks post-implantation. Data are expressed as the mean ± SD; ^*^P < 0.05 versus SCI group; ^#^*P* < 0.05 versus DSC + GH + bFGF group; ^NS^*P* > 0.05; *n* = 6.

The BBB score [37] was used to evaluate the rat hindlimb movement weekly for 8 weeks after SCI (Fig. 7A). No significant difference was found between the GH + bFGF and SCI groups (SCI: 1.75 ± 1.44 versus GH + bFGF: 3.17 ± 1.13, *P* > 0.05), and the DSC, DSC + GH, and lineage-specific matrix (DSC + GH + bFGF) groups showed significantly higher BBB scores compared to the SCI group (*P* < 0.05, Fig. 7A). Notably, the BBB scores further increased in the lineage-specific matrix group compared with the DSC group (*P* < 0.05), whereas the DSC + GH group did not (*P* > 0.05). These findings suggest that topographical and biochemical cues within the lineage-specific matrix work synergistically to promote motor rehabilitation after SCI. At the end of the 8 weeks, rats in the SCI group primarily exhibited passive limb dragging. By contrast, rats implanted with the lineage-specific matrix regained active dragging ability, achieved plantar placement in a weight-bearing stance, and exhibited occasional weight-bearing walking. Representative hindlimb movements for the lineage-specific matrix and SCI groups are shown in Movies S2 and S3.

To objectively assess spinal cord electrophysiological function, muscle motor evoked potentials (mMEPs) were measured to evaluate the transmission speed and amplitude of cortical signals passing through the lesion site at 8 weeks post-injury/implantation (Fig. 7B). The results revealed no significant difference in mMEP latency among the groups (*P* > 0.05, Fig. 7D). Compared with the SCI group, the GH + bFGF group showed no significant difference in mMEP amplitude (*P* > 0.05, Fig. 7C), while mMEP amplitude in DSC, DSC + GH, and lineage-specific matrix groups was significantly increased (*P* < 0.05). Notably, the lineage-specific matrix group exhibited a further increase in mMEP amplitude compared with the DSC group (*P* < 0.05), whereas the DSC + GH group did not (*P* > 0.05). These results suggest that the lineage-specific matrix improves cortical motor signal transmission through its synergistic topographical and biochemical effects.

Gait analysis was conducted at 8 weeks after injury/implantation to further evaluate motor recovery. Only rats in the lineage-specific matrix group met the system’s detection criteria. As shown in Fig. 7E, the footprint of a normal rat during ambulation demonstrates full weight bearing across the paw and toes, with the paw exhibiting the greatest load-bearing intensity. By contrast, the footprint of the rats in the lineage-specific matrix group (Fig. 7F) showed that they could actively drag their limbs, with weight bearing mainly concentrated in the palm and big toe. Compared to the coordinated step sequence of normal rats (Fig. 7G), the lineage-specific matrix group exhibited an uncoordinated step sequence (Fig. 7H) at 8 weeks post-implantation. Footprint intensity curves revealed that while normal rats distributed continuous and even pressure across both hindlimbs (Fig. 7I), rats in the lineage-specific matrix group exhibited uneven weight distribution (Fig. 7J). These findings suggest that while the hindlimb movements in the lineage-specific matrix group remained incomplete compared to normal rats, they demonstrated substantial gait recovery. Overall, these results indicate that the synergistic topographical and biochemical cues in the lineage-specific matrix enhance cortical motor signal transmission and promote motor recovery following SCI.

### The lineage-specific matrix promotes neural regeneration after SCI via integrin expression remodeling and AKT/ERK phosphorylation

**Fig. 8.**
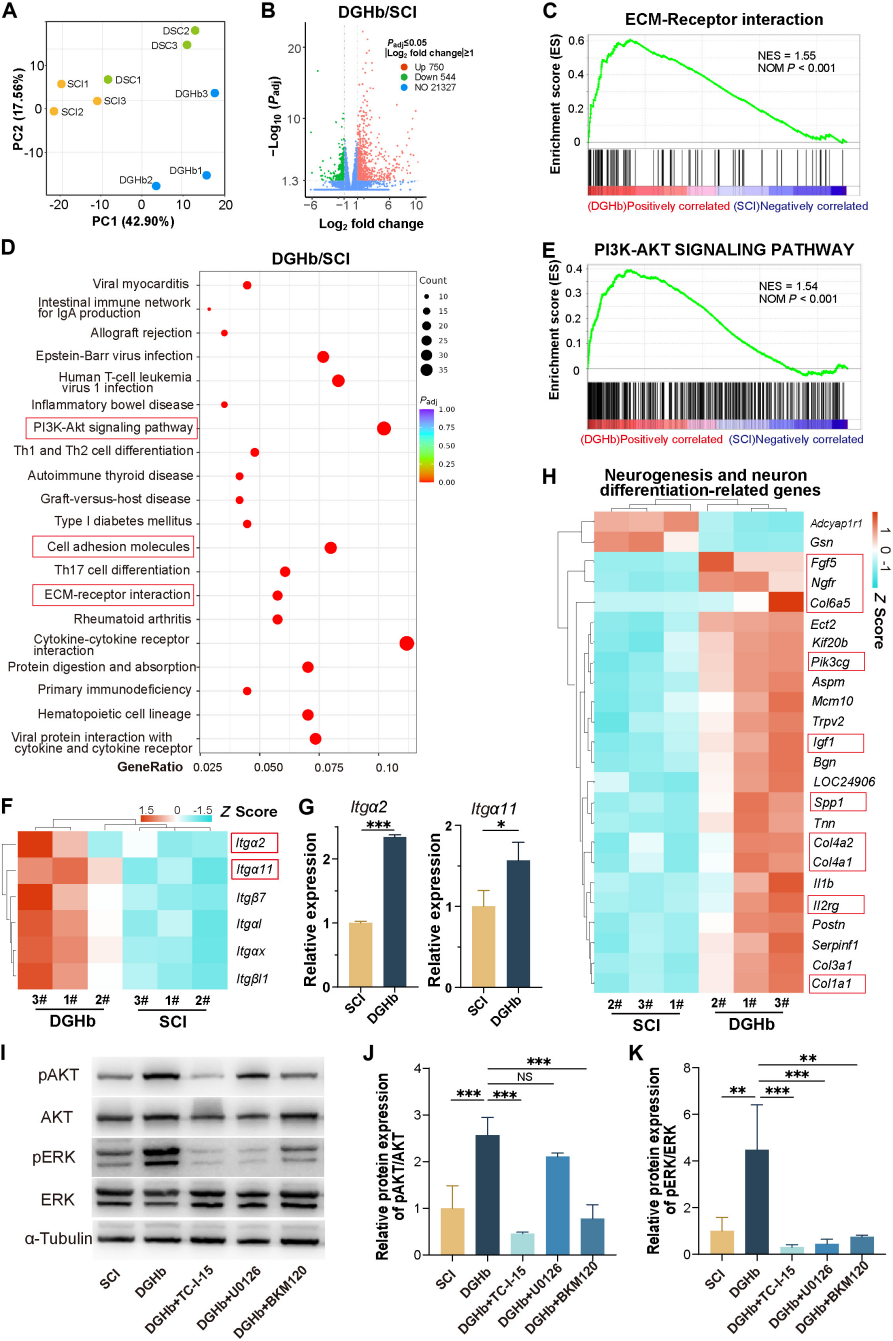
The lineage-specific matrix enhanced neural regeneration post-SCI through ITGA2/ITGA11–ERK/AKT axis. (A) PCA analysis of RNA sequencing showed gene expression patterns in the injury/graft area of the 3 groups (SCI, DSC, and lineage-specific matrix groups) at 2 weeks post-SCI. (B) The Volcano plot showed the up-regulated genes (red) and down-regulated genes (green) between the lineage-specific matrix (DGHb) and the SCI group. (C and E) GSEA analysis showed that genes related to ECM–receptor interaction and the PI3K–AKT signaling pathway were significantly enriched in the injury/graft area after lineage-specific matrix implantation. (D) The top 20 up-regulated KEGG pathways were listed based on the DEGs in the spinal cord tissues of the injury/graft area. (F) Expression profile heatmaps of ECM–receptor interaction-related integrin genes in the spinal cord tissues of the injury/graft area, as assessed by hierarchical clustering analyses. (G) qRT-PCR detection of Itgα2 and Itgα11 expression in the SCI and lineage-specific matrix groups 2 weeks post-SCI. (H) Heatmaps showed a selection of neurogenesis and neuronal differentiation-related genes from DEGs expressed in the spinal cord tissues of the injury/graft area, with the red box marking genes related to the PI3K–AKT signaling pathway. (I) Representative immunoblotting images showed the phosphorylation levels of AKT and ERK in the SCI group and lineage-specific matrix groups with or without different inhibitors at 2 weeks post-SCI. (J and K) The relative intensity of each band was normalized to their respective ɑ-tubulin loading controls, and the normalized p-AKT/AKT and p-ERK/ERK ratios were shown. Data are expressed as the mean ± SD; *P < 0.05, **P < 0.01, ***P < 0.001; *n* = 3.

To investigate the mechanism underlying SCI repair by the lineage-specific matrix, transcriptomic analysis was performed on the SCI/graft area at 2 weeks after implantation. PCA revealed distinct gene expression patterns between the SCI and lineage-specific matrix groups (Fig. 8A). A volcano plot showed that 750 genes were up-regulated, whereas 544 genes were down-regulated following lineage-specific matrix implantation (Fig. 8B). These results indicated that the lineage-specific matrix induces substantial gene expression changes. GSEA identified significant enrichment of genes associated with ECM–receptor interactions, focal adhesion, and the phosphatidylinositol 3-kinase (PI3K)–AKT signaling pathway (Fig. 8C to E and Fig. S5A).

To compare the detailed gene expression patterns between the SCI and lineage-specific matrix groups, differentially expressed genes (DEGs) were identified using DESeq2. The analysis revealed the top 20 up-regulated Kyoto Encyclopedia of Genes and Genomes (KEGG) pathways, including significant up-regulation in pathways such as ECM–receptor interaction, cell adhesion molecules, and the PI3K–AKT signaling pathway (Fig. 8D). To comprehend the differences among KEGG pathways in the alteration of adhesive molecules (ECM–receptor interaction, focal adhesion), related integrin expression patterns and downstream critical kinase activities were investigated for the SCI and lineage-specific matrix groups. To investigate integrin-mediated signaling, a heatmap analysis of integrin expression showed notable up-regulation of integrins *Itgα2* and *Itgα11* in the lineage-specific matrix group (Fig. 8F and G). Notably, these 2 integrins form heterodimers with Itgβ1, which are known to regulate cell proliferation and migration [38]. This observation suggested that lineage-specific matrix implantation activates a specific integrin-mediated signal transduction pathway.

Consistent with The lineage-specific matrix facilitates NSC migration, neuronal differentiation, and cell proliferation after SCI section, which demonstrated that the lineage-specific matrix enhances the migration, proliferation, and differentiation of endogenous NSCs after SCI, we further analyzed DEGs related to neural precursor cell proliferation (GO:0061351), neuroblast migration (GO:0097402), and positive regulation of neuron differentiation (GO:0045666). RNA-sequencing heatmap analysis (Fig. 8H) revealed significant up-regulation of genes associated with the PI3K–AKT signaling pathway, such as *Pik3cg*, *Spp1*, and *Ngfr*. The reliability of the transcriptome analysis was confirmed using quantitative reverse transcription polymerase chain reaction (qRT-PCR) (Fig. S5B). To validate these transcriptomic findings, we examined the effects of the biochemical components of the lineage-specific matrix, primarily bFGF and DSC, on the key signaling regulators AKT and ERK in NSCs in vitro (Fig. S5C and D). Western blot analysis revealed that bFGF induced transient ERK and AKT phosphorylation in NSCs at 10 min compared to the control group, although this effect diminished rapidly (Fig. S5E and F). By contrast, DSC induced sustained ERK and AKT phosphorylation, peaking at 30 min to 3 h before gradually decreasing (Fig. S5E and F). Taken together with the phenomena described in the in vitro evaluation (Lineage-specific matrix enhances the neurogenic niche of DSC scaffolds section), the lineage-specific matrix may enhance neuronal lineage differentiation by activating ERK and AKT phosphorylation.

Furthermore, Western blot analysis of proteins from the SCI/graft area at 2 weeks after implantation confirmed that lineage-specific matrix implantation significantly enhanced ERK and AKT phosphorylation (Fig. 8I). This biological effect was effectively inhibited by suppressing either the ITGA2/ITGA11, the PI3K–AKT, or the mitogen-activated protein kinase (MAPK)–ERK signaling pathways. Statistical analysis revealed that the pAKT/AKT and pERK/ERK ratios were significantly higher in the lineage-specific matrix group than in the SCI group, while these ratios were significantly decreased with the addition of the Itgα2/Itgα11 inhibitor (*P* < 0.05, Fig. 8J and K). Inhibition of the downstream PI3K–AKT or MAPK–ERK signaling pathways attenuated the phosphorylation of AKT or ERK in the injured area, respectively. These results indicate that the lineage-specific matrix promotes neural regeneration after SCI by activating ERK and AKT phosphorylation through integrin remodeling (*Itgα2*/*Itgα11*) and signaling pathway activation.

## Discussion

SCI often results in severe functional impairments, necessitating effective repair strategies [5]. Inspired by the ECM structure, this study used electrostatic adsorption and 3D printing to create a “core–shell” design that combines DSC and a hydrogel with controlled-release factors, forming a lineage-specific matrix with topographical and biochemical cues to enhance neurogenesis. Supported by a robust hydrogel shell, this matrix preserved the topological structure and biochemical composition of DSC while improving its proliferation properties. The core–shell design, consisting of a DSC-derived ECM core and a 3D conduit hydrogel shell, introduced novel topographical cues. The soft, flexible ECM core underwent microscopic deformation, modifying local mechanical properties and binding site density, thereby optimizing NSC adhesion in the local area [39]. Meanwhile, the rigid hydrogel shell provided structural support and served as an anchor for endogenous NSC migration, guiding neural regeneration on a macroscopic scale. Concurrently, the 3D conduit hydrogel shell guides endogenous neural regeneration at the macro level. Our results demonstrated that these instructive cues synergistically promoted NSC migration, proliferation, and neuronal differentiation by activating the ITGA2/ITGA11–ERK/AKT axis. Additionally, they modulated microglial activation, promoting M2 polarization and reducing the M1 phenotype associated with secondary injury. By establishing a neuronal lineage-specific environment conducive to endogenous neurogenesis, this lineage-specific matrix holds marked potential for SCI repair, ultimately enhancing functional recovery.

Decellularization alters ECM protein composition and concentration in the CNS, imparting novel properties that influence neural regeneration. The adult CNS ECM typically inhibits neural repair due to the presence of CSPGs and tenascins, which promote glial scar formation [22]. In this study, the DSC scaffolds, created through freeze–thaw and chemical extraction, were shown to facilitate neural regeneration. Proteomic analysis revealed an increase in neural-permissive proteins such as collagen, fibronectin, and laminin, alongside a reduction in inhibitory proteins such as CSPGs. This reorganization fostered a more favorable ECM environment for repair. For example, the 3-dimensional (3D) arrangement of laminin in DSC scaffolds guided directional axon growth through integrin α receptor interactions [14]. We found that DSC further modulated NSC behavior by altering integrin expression profiles and influencing AKT/ERK signaling pathways. Additionally, DSC implantation reduced M1-polarized microglia while promoting M2 polarization, supporting axonal regeneration [15,40]. These findings suggest that the ECM composition of DSC plays a crucial role in creating a regenerative microenvironment. Despite advancements in decellularization techniques, challenges persist in selectively preserving key ECM components [13]. Notably, soluble factors often exhibit greater alterations compared to structural proteins following decellularization [15,19,41,42]. Studies have indicated that DSC scaffolds enriched with soluble factors such as bFGF better support NSC proliferation than peripheral nerve scaffolds [19]. Our modified decellularization approach successfully retained beneficial growth factors, with bFGF levels comparable to those in fresh spinal cord tissue (Fig. 2K). Therefore, while DSC biochemical components enhance neural regeneration via ECM remodeling, further refinements are needed for clinical application.

In this study, we further enhanced the biochemical components of DSC by supplementing secreted factors. Together with the biochemical components of DSC, these factors constitute the biochemical cues of the lineage-specific matrix. As previously noted, the decellularization process results in the loss of beneficial secreted factors, thereby requiring supplementation [15,19,41]. Following a comparative analysis of decellularized scaffolds derived from neonatal and adult spinal cords, bFGF was selected as a growth factor to improve adult DSC in this study. This selection was particularly driven by proteomic data showing its higher retention in neonatal DSC [20], which is critical for neural cell proliferation. Our study found that while DSC alone did not significantly enhance cell proliferation in the injured region, the hydrogel shell with sustained-release bFGF significantly improved DSC’s proliferative effects in vivo. By integrating growth factors into adult DSC scaffolds, we more closely mimicked the neonatal ECM, creating an optimized environment for neuronal-specific regeneration.

Decellularized scaffold provides a neurogenic niche for neural tissue repair, which is also related to its complex topology [43]. Electron microscopy revealed that DSC exhibits a porous network of microfilaments and sheets that support NSC adhesion and migration. Preserving this topological structure is crucial for neuronal lineage-specific regeneration in vivo [19]. However, the mechanical strength of DSC significantly decreases upon wetting, resulting in collapse and deformation post-implantation, thereby disrupting its favorable topology [18,44]. To address this, we incorporated a robust hydrogel shell to maintain the DSC’s topological structure. To minimize hydrogel infiltration into the interior of the DSC and affect its topology, 3D printing was employed to precisely control the hydrogel thickness. The co-incubation and UV photocrosslinking conditions were adjusted to manage the infiltration. By optimizing hydrogel composition and synthesis, we developed a lineage-specific matrix with mechanical properties similar to those of native spinal cord tissue, which may support injury repair [45–47]. According to electron microscopy results, the lineage-specific matrix preserved the natural topological structure of DSC. Our experiments confirmed that the robust shell supported this topological structure in vivo and protected the graft area from fibrous scar invasion during the critical post-injury period, while the ECM-derived matrix with topographic cues promoted NSC proliferation and endogenous neuronal regeneration following SCI.

This study found that lineage-specific matrices not only promoted microglia to shift to the M2 type in the injury/graft area but also resulted in 12% of newborn cells differentiating into astrocytes, which play a crucial role in maintaining oxidative homeostasis in the CNS [48,49]. Given that glial scar formation can limit inflammatory spread and reduce the detrimental effects of fibrotic tissue and macrophages post-SCI, complete elimination of the scar may not be beneficial for SCI repair [48,49]. Following SCI, astrocyte levels increase from 36% to 43% during the acute phase but decline to approximately 4% by day 38, concurrently with a microglial increase from 18% to 71% [8]. Although the optimal astrocyte proportion and phenotype for SCI repair remain undefined, studies indicate that moderate reduction in glial scar density can enhance axon regeneration and functional recovery [8,49,50]. While this study observed no significant difference in astrocyte signals between the lineage-specific scaffold and SCI groups, a reduction in glial scar area was evident. Although functional neuronal relay improved 8 weeks post-injury in this study, further research is necessary to optimize the temporal and spatial dynamics, as well as the coordinated regeneration of multiple neural lineages, for enhanced SCI repair.

This study verified the synergistic effects of topographical and biochemical cues on SCI recovery. While previous research has shown that either DSC’s topological structure or its biochemical components can partially promote neural regeneration post-SCI, achieving effective neural relay and motor rehabilitation remains challenging [18,25,28,41]. Therefore, combination therapy is necessary for effective neurogenesis [1,51]. Our lineage-specific matrix facilitated SCI repair through multiple mechanisms. First, it enhanced the regenerative microenvironment by promoting M2-polarized microglia in the injured or grafted area. Second, it activated ERK/AKT phosphorylation by remodeling the integrin expression profile and up-regulating the PI3K–AKT signaling pathway. Finally, it improved cortical signal transmission through the injury site, leading to improved motor function in SCI rats. These findings suggest that our combination therapy strategy not only promotes neuronal regeneration but also establishes an effective neural relay for motor rehabilitation.

This study developed a lineage-specific matrix for SCI repair using additive manufacturing of natural decellularized extracellular matrix (dECM), demonstrating strong translational potential [52]. Our approach combined 3D printing with photochemical crosslinking technology to achieve precise control over matrix structure and properties, facilitating scalable production. Furthermore, the matrix can be patient-specific, customized based on MRI scans of the DSCs and spinal lesions using computer-aided design (CAD) software [29,53,54]. Despite these advances, several challenges remain for preclinical translation, including the adoption of safer decellularization methods such as supercritical carbon dioxide (scCO₂) extraction and the evaluation of animal-derived dECM sources for large-scale scaffold production [21]. To ensure consistent and safe scaffold manufacturing, establishing a standardized, Good Manufacturing Practice (GMP)-compliant decellularization process is essential to minimize batch-to-batch variations [55]. Additionally, safety, sterilization, and preservation protocols must meet regulatory standards for clinical dECM application, addressing critical factors such as decellularization efficacy, chemical residue clearance, ECM composition integrity, and scaffold sterility [52]. Although promising for SCI repair, extensive preclinical evaluation—including in vitro assays and in vivo primate studies—is required prior to human trials to assess toxicity, microbial safety, immunogenicity, functionality, and biocompatibility [21,29,52]. Finally, clinical implementation of the final biological scaffold will necessitate the establishment of GMP-compliant facilities, cleanroom environments, equipment, qualified personnel, monitoring systems, and packaging [55].

In conclusion, we developed a lineage-specific matrix that enables neuronal-specific regeneration of endogenous NSCs in the injury/graft area, ultimately facilitating the formation of functional neuronal relays post-SCI. The hydrogel shell provided mechanical support, maintaining the DSC’s topological structure and enhancing NSC migration and axon growth. Moreover, the controlled release of bFGF improved the proliferation properties of adult DSC, mimicking the neonatal spinal cord ECM. This structure-inspired lineage-specific matrix thus creates a mechanically compatible and biologically optimized environment that effectively promotes endogenous stem cell recruitment, neuronal differentiation, and axonal regeneration by activating the ITGA2/ITGA11–ERK/AKT axis. The result is significant motor function improvement in SCI rats. Our findings highlight the lineage-specific matrix as a promising strategy for SCI treatment, integrating instructive topographical and biochemical cues to enhance recovery outcomes.

## Methods

### Ethics statement

All animal experiments were conducted in compliance with the Guide for the Care and Use of Laboratory Animals from the Army Military Medical University, adhering to the protocols approved by the local animal ethics committee (AMUWEC20224973).

### Preparation of DSC

Sprague–Dawley rats (220 to 250 g, provided by the Experimental Animal Center of Army Medical University, China) were anesthetized using 1% pentobarbital sodium (50 mg kg^−1^, intraperitoneally). The thoracic spinal cord (T4 to T13), approximately 2 cm in length, was aseptically dissected, immersed in precooled phosphate-buffered saline (PBS), and rinsed 3 times to remove surface blood cells. Subsequently, the spinal cord was sectioned into segments of approximately 1 cm each. Decellularization was then carried out using a combination of physical freeze–thaw cycles and chemical extraction, as described in the previous study [56]. In brief, after being frozen overnight at −80 °C, the samples were thawed at room temperature. Three cycles were conducted, each including 7 h in distilled H_2_O (dH_2_O), 14 h in 4% (w/v) sodium deoxycholate (D6128, Macklin, China), 15 min in PBS, 1 h in deoxyribonuclease (DNase) I (0.1 mg ml^−1^, Roche, 11284932001), 15 min in PBS, 4 h in dH_2_O, 2 h in 3% (v/v) Triton X-100 (ZLI9308, ZSGB-BIO, China), 15 min in PBS, 1 h in DNase I, and 15 min in PBS. After all cycles, the scaffolds were soaked in dH_2_O for 1 h, during which the dH_2_O was changed 5 times. All reagents contained 1% penicillin and streptomycin, and all procedures were conducted under sterile conditions. All steps were performed on a shaker at 120 rpm at room temperature. Finally, the DSC scaffolds were then soaked in dH_2_O, freeze-dried, and stored at −80 °C.

### Preparation of GH hydrogels

All GH hydrogels were synthesized through free radical copolymerization by combining GelMA (50 mg) powder, lithium phenyl(2,4,6-trimethylbenzoyl)phosphinate (LAP; 2.5 mg), and varying quantities (G_5_H_0_: 0 mg, G_5_H_0.25_: 2.5 mg, G_5_H_0.5_: 5 mg, G_5_H_1_:10 mg) of methacrylated heparin (HepMA) in the PBS solution (1,000 μl). In addition, to better distinguish the hydrogel from the DSC, 5 mg of blue alkene-coupled fluorescent dye powder (EFL-DYE-UF-ENE-B, EFL-Tech, China) was added to G_5_H_0.5_ pregel solution (1,000 μl) to prepare blue fluorescent hydrogel. The mixture was dissolved under dark conditions, agitated, and sterilized using a 0.22-μm sterile needle filter to produce the GH pregel solution. This pregel solution was then aspirated and transferred into a cylindrical transparent polypropylene mold, followed by irradiation with a 365-nm light-emitting diode (LED) light for 60 s to induce gelation. Subsequently, the hydrogel was immersed in PBS and stored under refrigeration until further use.

### Fabrication of the lineage-specific matrix

The dissolved G_5_H_0.5_ pregel solution was aspirated into a 1-ml syringe and allowed to stand until the bubbles disappeared. The hydrogel shell was modeled as a round tube with an outer diameter of 3 mm and an inner diameter of 2.5 mm, and was printed using a 3D bioprinter (LivPrint 3D Bioprinter, China). The length of the shell was adjusted to 5 to 10 mm, corresponding to the DSC length. The printing parameters were set as follows: layer height of 0.2 mm, printing speed of 1.5 mm s^−1^, nozzle diameter of 0.19 mm, and a printing temperature of 4 °C. The freeze-dried DSC scaffold was inserted into the pregel shell and subsequently wetted by the addition of PBS. It was then heated to 37 °C within the mold. Following the melting process, the pregel shell was immediately exposed to LED light at a wavelength of 365 nm for 60 s to induce gelation. All procedures were conducted under aseptic conditions. Furthermore, the lineage-specific matrix (DSC + G_5_H_0.5_ + bFGF) was prepared by incubating the DSC + GH (DSC + G_5_H_0.5_) scaffolds with bFGF solution (1 μg ml^−1^, Cloud-Clone Corp., China) at 37 °C for 5 h.

### Compressive modulus test

The compressive modulus of the fresh rat thoracic spinal cord, DSC, G_5_H_0.5_ + bFGF, and DSC + G_5_H_0.5_ + bFGF groups was assessed using the CMT6103 universal testing system (MTS Corp., USA). For the DSC + G_5_H_0.5_ + bFGF group, the compression modulus was evaluated prior to implantation and subsequently at 1, 2, and 4 weeks following implantation. In brief, scaffolds with dimensions of 3 mm in diameter and 3 mm in thickness were subjected to testing at a rate of 0.5 mm min^−1^. The compressive modulus was determined from the stress–strain curve. Before the measurement, the scaffolds were soaked with PBS solution to simulate the in vivo environment.

### Cryo-scanning electron microscopy

The morphology of the DSC, G_5_H_0.5_, and DSC + G_5_H_0.5_ + bFGF groups was analyzed using cryo-SEM. Initially, the samples were fixed with 2.5% glutaraldehyde and subsequently immersed in liquid nitrogen at −210 °C to achieve low-temperature vitrification. The samples underwent a sublimation process for 10 min prior to gold coating. Following this preparation, the samples were transferred to the SEM sample chamber, where the voltage was adjusted to identify suitable regions for image acquisition. The porosity of the scaffold was evaluated by examining 5 randomly selected fields at a magnification of 1,000×. In the DSC + GH + bFGF group, visual fields were randomly selected within the central DSC area to assess porosity. Finally, the selected regions were analyzed in ImageJ software (Wayne Rasband, National Institutes of Health, USA).

### Proteomics

Adult male rat (220 to 250 g) spinal cord and DSC proteins were extracted for proteomics detection (*n* = 3 for each group). The sample was grinded with liquid nitrogen into cell powder, followed by lysis buffer, sonication, and centrifugation. The supernatant was then collected, and protein concentrations were determined using the BCA kit according to the manufacturer’s instructions. The 4D label-free quantification (LFQ) proteomics analysis, including trypsin digestion, liquid chromatography–tandem mass spectrometry (MS/MS), and data analysis, was conducted by Jingjie PTM BioLabs. Peptide samples were dissolved in 2% acetonitrile/0.1% formic acid after trypsin digestion and separated using a homemade reversed-phase column (25 cm length, 100 μm inner diameter). The nanoElute UHPLC system (Bruker Daltonics) and timsTOF Pro MS were used for separation and analysis. MS/MS data were processed with the DIA-NN search engine (v.1.8). Tandem mass spectra were matched to the Rat UniProt database (47,943 entries) combined with a reverse decoy database. Differentially expressed proteins were identified by calculating the average fold change between biological replicates. Proteins with fold change > 1.5 and *P* < 0.05 were considered to be significantly differentially expressed.

### In vitro bFGF release from the GH hydrogel

The study used a composite hydrogel made of GelMA and HepMA, with HepMA facilitating the bFGF adsorption through electrostatic interactions. The sustained release of bFGF from the composite hydrogels was evaluated using ELISA. Initially, GH hydrogel cylinders with varying concentrations (G_5_H_0_, G_5_H_0.25_, G_5_H_0.5_, G_5_H_1_) were prepared, each with a diameter of 5 mm and a thickness of 3 mm. These samples were subsequently placed in 24-well plates (ultra-low attachment surface, Corning Incorporated) and immersed in 1 ml of PBS. Following the removal of unabsorbed water, 1 μg ml^−1^ bFGF solution (Cloud-Clone Corp., China) was added at a hydrogel to bFGF volume ratio of 3:10. The samples were then incubated for 5 h at 37 °C. At designated time intervals, 1 ml of the buffer was extracted and stored at −20 °C, while 1 ml of fresh buffer was replenished. The cumulative sustained-release bFGF was quantified by ELISA kits (Cloud-Clone Corp., China).

### NSC isolation and 3D cell culture

Primary NSCs were isolated from E14 embryo as described in a previous study [57]. Briefly, pregnant Sprague–Dawley rats (supplied by the Experimental Animal Center of Army Medical University, China) were anesthetized with 1% pentobarbital sodium (50 mg kg^−1^, intraperitoneally). Embryos were extracted under sterile conditions, placed in precooled Dulbecco’s modified Eagle’s medium (DMEM)/F12 (Gibco, USA), and carefully stripped of the leptomeninges, and the hippocampus tissue was cut into pieces. Then, Accutase solution (Gibco, USA) was added and incubated at 37 °C for 5 min. After discarding the digestion solution, NSC proliferation medium (DMEM/F12 + 2% B27 + 1% glutamax + 20 ng ml^−1^ epidermal growth factor + 20 ng ml^−1^ bFGF, Gibco, USA) was added, and the cell suspension was filtered through a 25-μm mesh. After counting the filtered NSC suspension, the cells were cultured in T75 culture flasks and placed in an incubator.

In vitro 3D cell culture was performed on the scaffold to assess the cytocompatibility of the scaffold. For each hydrogel group (G_5_H_0_, G_5_H_0.25_, G_5_H_0.5_, G_5_H_1_), GH pregel solution (30 μl) was initially dropped into the glass bottom of a cell culture dish (15-mm glass bottom cell culture dish, NEST) and exposed to 365-nm LED light for 60 s to induce gelation. Subsequently, bFGF solution (100 μl, 1 μg ml^−1^) was added and incubated at 37 °C for 5 h, followed by 3 washes with PBS. For the DSC and DSC + GH + bFGF group, the scaffold was sectioned to a thickness of 100 μm before in vitro culture. Then, cell suspensions were centrifuged at 1,000 rpm for 5 min and resuspended in the appropriate medium. Among them, 100 μl of C17.2 (5 × 10^4^ cells ml^−1^) cell suspensions was dripped slowly to each scaffold and cultured in DMEM (Gibco, USA) with 10% fetal bovine serum (Gibco, USA). Separately, 50 μl of NSCs (1 × 10^7^ cells ml^−1^) was seeded on scaffolds and cultured in the NSC proliferation medium to assess cell viability or in the differentiation medium (DMEM/F12 + 2% B27 + 1% glutamax, Gibco, USA) to assess differentiation. Finally, the scaffolds were incubated at 37 °C under 80% to 90% humidity, and the culture medium was refreshed after 2 h and every other day.

### In vitro distribution, survival, proliferation, and differentiation of cells

To effectively show the cellular distribution on scaffold, hydrogel in the DSC + GH + bFGF group was labeled with blue fluorescent dyes. After 1 and 7 d of culture, NSC cells in each group (G_5_H_0.5_ + bFGF, DSC, DSC + GH + bFGF) underwent live/dead staining (C2015M, Beyotime, China) to assess cellular distribution on the scaffolds. In addition, the C17.2 cells in each group (G_5_H_0_ + bFGF, G_5_H_0.25_ + bFGF, G_5_H_0.5_ + bFGF, G_5_H_1_ + bFGF, DSC, DSC + GH + bFGF) underwent live/dead staining to evaluate cell viability on the scaffolds at 1, 3, and 5 d post-seeding (*n* = 6). The survival of NSC on the scaffolds (G_5_H_0.5_ + bFGF, DSC, DSC + GH + bFGF) was also assessed at 1, 3, and 7 d post-seeding. The procedure is as follows: A mixture of 1 μM Calcein-AM and 1 μM EthD-III was added to the hydrogel and incubated at 37 °C for 50 min. The hydrogel was then washed 3 times with D-Hank’s solution and examined under a fluoroscope. Green fluorescence indicated live cells, and red fluorescence indicated dead cells. The cell survival rate was determined by dividing the number of green fluorescent cells by the total number of fluorescent cells.

The cells in each group (G_5_H_0_ + bFGF, G_5_H_0.25_ + bFGF, G_5_H_0.5_ + bFGF, G_5_H_1_ + bFGF, DSC, DSC + GH + bFGF) were subjected to EdU staining (C0071S, Beyotime, China) to assess cell proliferation on the scaffolds (*n* = 6). Briefly, after 3 d in 3D culture, 10 μM EdU was added and incubated for 3 h. Cells were then fixed with 4% paraformaldehyde (PFA) for 30 min, permeabilized with 0.3% Triton X-100 for 15 min, incubated with the Click Reaction Mixture for 30 min in the dark at room temperature, and stained with DAPI for 10 min. Proliferation rate was determined by the ratio of EdU-labeled to DAPI-labeled cells.

After 7 d of culture, cells in each group (G_5_H_0.5_ + bFGF, DSC, DSC + GH + bFGF) were fixed using 4% PFA and subsequently subjected to immunofluorescence staining. To evaluate the differentiation of NSCs on the scaffolds (*n* = 5), Tuj1 (ab18207, 1:1,000, Abcam, UK) and GFAP (BM0055, 1:100, Boster Bio, China) were employed to label NSC differentiated neurons and astrocytes, respectively.

### Immunogenicity test of the scaffold

The immunogenicity of bioscaffolds was assessed by implanting the bioscaffolds into the muscles. After adult Sprague–Dawley rats (*n* = 5, 220 to 250 g) were anesthetized with 1% pentobarbital sodium (50 mg kg^−1^, intraperitoneally), 4 groups of bioscaffolds (gelatin sponge, G_5_H_0.5_ + bFGF, DSC, DSC + GH + bFGF) with lengths of 3 mm were implanted into the paravertebral muscles. The incisions were subsequently sutured, and levofloxacin lactate (10 mg kg^−1^ day^−1^, intraperitoneally) was administered daily for 3 d post-injury/implantation. Seven days following implantation, all rats were re-anesthetized with 1% sodium pentobarbital and subjected to transcardiac perfusion with 0.1 M PBS and 4% PFA. The tissues containing bioscaffolds were sectioned to a thickness of 30 μm, and macrophage infiltration within the graft region was evaluated via immunofluorescence analysis. Iba1 was employed to label macrophages in the graft area, while CD68 was utilized to identify activated macrophages. The immunogenicity of the bioscaffolds was assessed by quantifying the number of cells double-positive for CD68 and Iba1 across 5 randomly selected fields.

### Scaffold implantation

SCI model animals were prepared as described in the previous study [58]. The adult male Sprague–Dawley rats (220 to 250 g) were randomly allocated into 5 groups, with 10 rats in each group. The groups were as follows: the SCI group, no scaffold was implanted; the GH + bFGF group, implanted G_5_H_0.5_ hydrogel with bFGF modification; the DSC group, implanted DSC; the DSC + GH group, DSC coated with G_5_H_0.5_ hydrogel shell was implanted; the DSC + GH + bFGF group, implanted DSC + GH scaffolds with bFGF modification.

During surgery, rats were anesthetized with 1% sodium pentobarbital (50 mg kg^−1^, intraperitoneally) and underwent laminectomy at T8 to T10 under sterile conditions. Using an operating microscope, a 2-mm section of the spinal cord was removed at T8 to T10 with angled microscissors. Following retraction of the stumps, a 2.5-mm gap was established, into which scaffolds were implanted in all experimental groups except for the SCI group. Subsequent to the suturing of the surgical incision, the experimental rats were provided with comprehensive post-operative care. This included the administration of intraperitoneal injections of levofloxacin lactate (10 mg kg^−1^ day^−1^) for 5 d, alongside manual bladder voiding performed twice daily for 4 weeks.

The experimental rats were anesthetized with 1% sodium pentobarbital at 1 or 8 weeks and then transcardially perfused with cold 0.1 M PBS and 4% PFA. The spinal cord was dissected, fixed in 4% PFA for 24 h at 4 °C, and placed in 10%, 20%, and 30% phosphate-buffered sucrose solutions for 24 h each. After freezing, samples were embedded in optimal cutting temperature compound (SAKURA FINETEK, USA) and the thoracic spinal cords were sectioned longitudinally to 20-μm thickness. For sample sectioning, a longitudinal section was obtained at 100-μm intervals for subsequent staining.

### 3D morphological evaluation of scaffolds in vivo

Initially, the 3D morphology of the lineage-specific matrix in vivo was evaluated by MRI (Aspect M7, Israel). The T2-weighted (T2W) image technique allows for the visualization of hydrogels without using contrast agents. MRI T2W scans in axial, sagittal, and coronal planes were conducted on rats’ spinal cord implantation sites at 1, 2, 3, 4, and 8 weeks post-implantation of the lineage-specific matrices. MRI scanning parameters were as follows: slice thickness, 1.2 mm; interslice gap, 0.2 to 0.25 mm. The outer contour circularity and cross-sectional area of the lineage-specific matrix in MRI axial planes were analyzed with ImageJ.

Then, to assess the degradation of hydrogel shells in the graft area, the DSC + GH + bFGF scaffolds labeled with blue fluorescence were implanted into the spinal cords of male Sprague–Dawley rats (*n* = 8, 220 to 250 g). One rat was randomly sacrificed every week until 8 weeks after operation, and the spinal cords containing the scaffolds were dissected for immunofluorescence staining to evaluate the degradation of the hydrogel shell. To improve the distinction between the rat spinal cord and the hydrogel shell, an anti-rat secondary antibody was utilized for direct labeling of the rat spinal cord.

### In vivo EdU labeling and detection

EdU, a thymidine analog used for labeling newly synthesized DNA, was employed to evaluate cell proliferation in vivo. EdU (ST067, Beyotime, China) was administered to rats (4 mg kg^−1^, intraperitoneally, *n* = 6) at 3 dpi and subsequently on a weekly basis. At 8 weeks post-injury/implantation, EdU-labeled cells within the lesion area of the spinal cord were identified using the EdU Cell Proliferation Kit (C0081S, Beyotime, China). Following EdU labeling, immunofluorescence staining for Tuj1 and GFAP was conducted to assess the neurogenic differentiation of newly proliferating cells within the lesion area in vivo.

### H&E staining and Masson’s trichrome staining

Masson’s staining, which is dependent on the permeability to anionic dyes of varying sizes, stains tissues such that mature scars rich in collagen fibers appear blue. H&E staining, as well as Masson’s trichrome staining, was conducted at the eighth week post-injury/implantation. In brief, the spinal cords were fixed in 4% PFA, followed by dehydration using a graded series of ethanol. The tissues were then cleared with xylene and subsequently infiltrated with molten paraffin. Longitudinal sections of the paraffin-embedded spinal cords were obtained at a thickness of 5 μm. The sections were stained using H&E or Masson’s trichrome staining protocols as previously described [58]. Finally, the stained slides were mounted with neutral resin and examined on Olympus VS200 microscope (Olympus, Japan). At magnifications of either 100× or 200×, 5 randomly selected fields within the injury/graft area were analyzed to evaluate spinal cord cavities and fibrous scar. The selected fields were quantified using ImageJ software (Wayne Rasband, National Institutes of Health, USA) as described in a previous study [58]. Spinal cord cavities were assessed via H&E staining by measuring the area of the cavities within the injury/graft region as well as within a 3-mm area on either side (cavities smaller than 2,500 μm^2^ were excluded). Fibrous scar was evaluated using Masson’s staining by determining the scar-to-tissue area ratio in selected fields.

### Immunofluorescence staining

Immunofluorescence staining was conducted as previously described [58] at 1 and 8 weeks post-injury/implantation. Briefly, frozen sections were fixed with 4% PFA for 30 min, permeabilized with 0.3% Triton X-100 for 30 min, and blocked with Blocking Buffer (P0220, Beyotime, China) for 30 min at room temperature. Subsequently, pre-diluted primary antibodies were added and incubated overnight at 4 °C. The sections were then rinsed with PBS and incubated with pre-diluted secondary antibodies for 2 h at room temperature. Nuclear staining was conducted utilizing DAPI staining solution (P0131, Beyotime, China). Finally, the slides were examined using laser scanning confocal microscopy (LSCM; Zeiss 780/880, Germany). A comprehensive list of all antibodies employed in this study, encompassing both primary and secondary antibodies, is provided in Table S1. At magnifications of either 100×, 200×, or 400×, 6 randomly selected fields within the injury/graft area were analyzed to evaluate hydrogel degradation, cell proliferation, and neural or immune cell invasion. Tuj1 and GFAP were assessed by calculating the ratio of immunofluorescence-positive areas to the selected fields. NSC migration in the injury/graft area was measured by the ratio of Nestin-positive cells to total DAPI-stained cells in the selected field. Cell proliferation in the injury/graft area was measured by counting EdU-positive cells or the ratio of EdU-positive to DAPI-positive areas. Neuronal or astroglial differentiation was evaluated by the percentage of Tuj1- or GFAP-positive cells among EdU-positive cells in selected fields. Activated microglia invasion in the lesion was evaluated by counting CD86 and Iba1 double-positive cells, while M2-polarized microglia invasion was assessed by counting CD206 and Iba1 double-positive cells in the selected fields.

### Behavior analysis

Hindlimb motor recovery was evaluated utilizing the BBB scale during the open field test. The BBB scale ranges from 0 to 21, where higher scores denote near-normal hindlimb movement. In each experimental group, 6 rats were randomly selected and allowed free movement within the open field. Two independent observers, blinded to the treatment assignments, observed each rat for 5 min. The experimental animals were observed every 7 d until 8 weeks after surgery. Furthermore, the gait analysis system (CatWalk XT, Noldus Information Technology, Netherlands) was employed to evaluate the step sequence and weight-bearing status of the hindlimbs. The step sequences were divided into Cruciate type (CA: RF-LF-RH-LH, CB: LF-RF-LH-RH), Alternate type (AA: RF-RH-LF-LH, AB: LF-RH-RF-LH), and Rotate type (RA: RF-LF-LH-RH, RB: LF-RF-RH-LH). This system required that the rats make palmar contact with the ground and traverse a specified distance during ambulation. Only the DSC + GH + bFGF group met these criteria; thus, measurements were exclusively conducted on this group, with normal rats serving as controls. The system provided an analysis of the load-bearing status, step sequence, and mean intensity of the corresponding footprints of the rats.

### Electrophysiology analysis

At 8 weeks after surgery, the action-evoked potentials of the rats were assessed using an EMG System (Keypoint Focus EMG System, Alpine Biomed, USA) (*n* = 6). Following administration of isoflurane anesthesia, a stimulating needle was inserted into the extracranial projection area of the rats’ motor cortex. The receptive electrode was positioned in the gastrocnemius muscle, while the ground electrode was placed in the lumbar paravertebral muscles. The stimulation was delivered at an intensity of 20 mA using a single pulse. To ensure the acquisition of a morphologically stable waveform, each rat underwent 5 stimulations, with a 1-min interval between each measurement prior to recording.

### Transcriptome analysis and qRT-PCR

Two weeks post-surgery, spinal cord tissue samples from the injury/implant sites were collected from 3 groups of rats (SCI group, DSC group, and lineage-specific matrix group) for transcriptomic sequencing (*n* = 3). In alignment with established methodologies [57], total RNA was isolated utilizing the TRIzol reagent (Sangon, China) in accordance with the manufacturer’s guidelines, followed by treatment with ribonuclease (RNase)-free DNase I to eliminate genomic DNA contamination. The integrity of the RNA was verified using a 1.0% agarose gel. Subsequently, RNA quality and concentration were determined using a NanoPhotometer spectrophotometer (IMPLEN, USA) and an Agilent 2100 Bioanalyzer (Agilent Technologies, USA). Library preparation was conducted with the Illumina NEBNext Ultra RNA Library Prep Kit, adhering to the manufacturer’s instructions. The experimental procedures were executed at Novegene Technology Co. Ltd. (Beijing, China). Sequencing reads were aligned to the rat reference genome using HISAT2. The expression levels of the samples were quantified using FPKM (fragments per kilobase of exon model per million mapped fragments). The expression levels of the entire probe set were normalized, and DEGs were identified using DESeq2 analysis, applying a fold-change threshold of 2 and a *P* value of less than 0.05. KEGG pathway enrichment analysis was conducted using the ClusterProfiler package. The Enrich-KEGG method was employed to perform enrichment testing for KEGG pathways, with a *P* value threshold of less than 0.05, which automatically calculates enriched functional categories for each gene cluster. Similarly, the Enrich-GO method was used to conduct enrichment testing for GO terms, also with a *P* value cutoff of less than 0.05.

Total RNA was extracted from spinal cord samples using the RNA Extraction Kit. Subsequently, cDNA synthesis was carried out via reverse transcription of the RNA. qPCR was conducted in accordance with the manufacturer’s protocol, utilizing the SYBR Premix Ex TaqII on the CFX96 System (Bio-Rad, USA). Gene expression levels were normalized to glyceraldehyde-3-phosphate dehydrogenase (GAPDH) and analyzed using the threshold method (*F* = 2−ΔΔCt). The sequences of the primers employed are provided in Table S2.

### Protein extraction and Western blot

The experiment involved 3 groups: a control group, a bFGF group, and a DSC group. The control group received mitogen-free NSC medium (DMEM/F12 medium containing 2% B27), the bFGF group was given medium with 20 ng/ml bFGF, and the DSC group received medium with 1 mg/ml DSC solution. To prepare the DSC solution, 5 mg of DSC powder was digested in 0.01 M HCl with 1 mg/ml pepsin for 24 h at 25 °C. After digestion, particulates were removed by centrifuging at 30,000 rpm for 30 min at 4 °C. The pH was adjusted to 7.4 with 1 M NaOH, and the solution was made isotonic with 10× PBS before being stored at −20 °C. Before seeding, neural stem/progenitor cell (NSPC) spheroids were starved for 12 h in mitogen-free medium, dissociated into single cells, and seeded onto poly-l-lysine-coated 6-well plates at 800,000 cells per well. After 12 h, the medium was replaced with either mitogen-free, bFGF-supplemented, or DSC-supplemented medium. At various intervals, cells were collected, washed with cold tris-buffered saline containing 0.1% Tween 20 (TBST), and lysed in radioimmunoprecipitation assay (RIPA) buffer with protease and phosphatase inhibitors for 30 min. The lysate was clarified by centrifugation, and protein quantification was done using a bicinchoninic acid (BCA) kit before mixing with loading buffer and undergoing thermal denaturation. For electrophoresis, protein samples (20 μg per lane) were loaded onto a sodium dodecyl sulfate–polyacrylamide gel, followed by immunoblotting on polyvinylidene fluoride membranes.

There were 5 groups (*n* = 3): the SCI group, the lineage-specific matrix group, and 3 treatment groups receiving the lineage-specific matrix with either TC-I-15 (an inhibitor of Itgα2 and Itgα11), BKM120 (a PI3K inhibitor), or U0126 [a MAPK kinase 1/2 (MEK1/2) inhibitor]. After implantation of the lineage-specific matrix, the TC-I-15 group received 0.55 mg/kg/day intrathecally, the BKM120 group received 20 mg/kg/day orally, and the U0126 group received 30 mg/kg/day intraperitoneally [59–63]. Two weeks post-surgery, p-AKT/AKT and p-ERK/ERK expression in rat SCI tissues was analyzed via Western blotting. For spinal cord tissues, they were frozen with liquid nitrogen and ground into powder using a freezer mill (Retsch MM400, Germany). A 10-mg tissue sample was lysed in 200 μl of pre-cooled RIPA buffer with inhibitors for 30 min. Subsequent steps followed a previously described protocol [57]. Details of antibodies used are in Table S1. Each Western blot was done in triplicate, and protein signal intensity was measured with ImageJ software.

### Statistical analysis

Data analysis was conducted using GraphPad Prism (GraphPad Software, Boston, USA). The means and standard deviations for each data group were evaluated through one-way analysis of variance (ANOVA). In cases where equal variances were confirmed, the least significant difference test was employed; otherwise, Dunnett’s T3 multiple comparisons test was utilized. A 2-sided *P* value of less than 0.05 was considered indicative of statistical significance.

## Data Availability

The data that support the findings of this study are available from the corresponding author upon reasonable request. Figures 1A, 3A, and 5A were created in BioRender, Bo Wu (2025). https://BioRender.com/uxonp1k.
