# Supplementary material for: Structure-Inspired Lineage-Specific Matrix for Endogenous Neurogenesis in Spinal Cord Injury
Source: Research (Wash D C). 2025 Aug 7;8:0821. doi: 10.34133/research.0821 (PMC12329214; doi:10.34133/research.0821)
Supplement: Supplementary 1 — Figs. S1 to S5 Tables S1 and S2 Movies S1 to S3 [file research.0821.f1.zip › supplementary figures -Clean Version.docx]

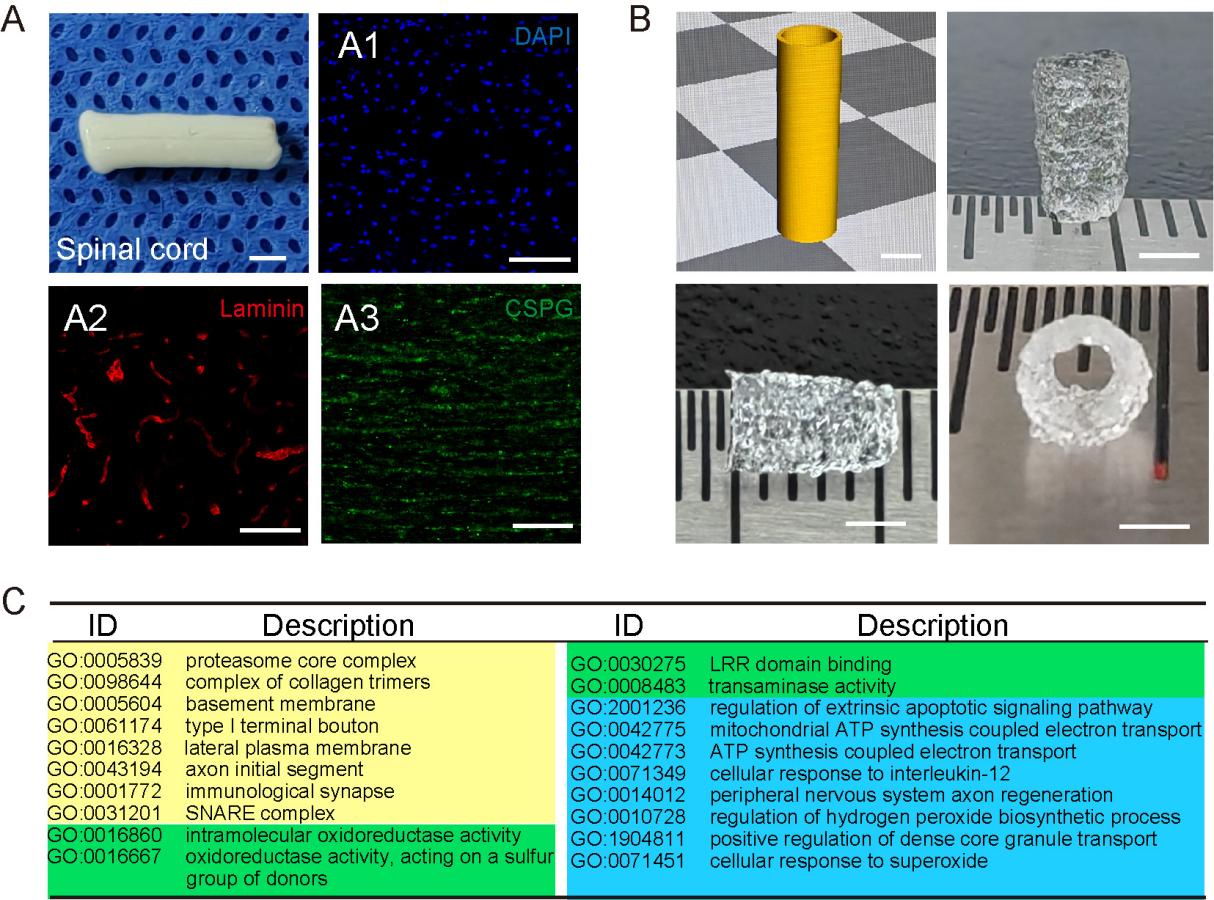


**Fig. S1: Supplementary characterization of the scaffolds.** (A) Images of fresh adult rat spinal cord and immunofluorescence of DAPI, Laminin, and CSPG. When compared with the adult spinal cord, DSC retained a large number of Laminin while removing most of the CSPG. (B) Design drawing and three views (front, side and top views) of the 3D-printed GH hydrogel shell. (C) GO pathway annotation in functional enrichment analyses of differential proteins in Fig. 1I. Scale bars = 2 mm in (A, B), 100 µm in (A1 - A3).


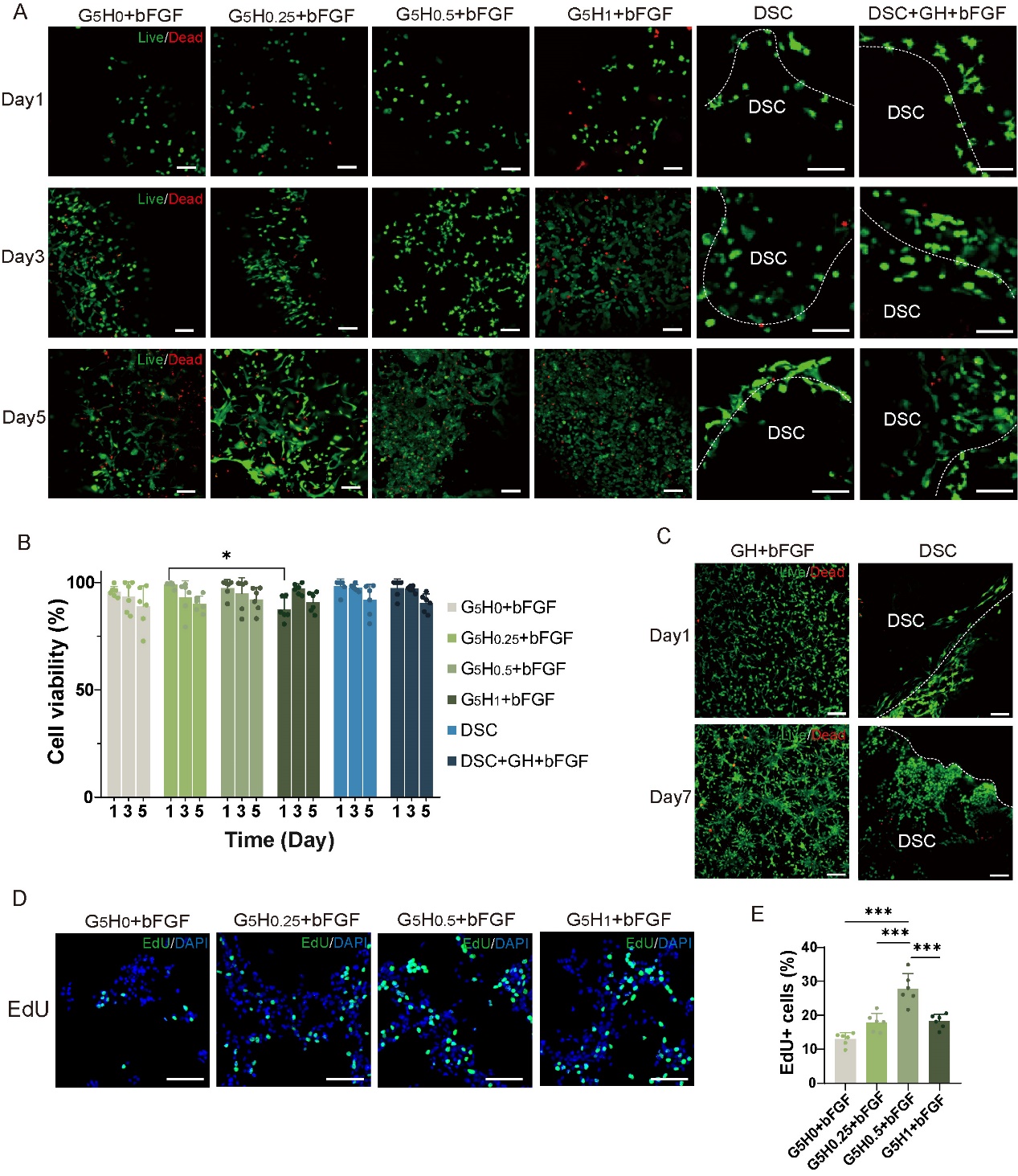


**Fig. S2: In vitro cell survival and proliferation in different proportions of composite hydrogels.** (A) Live/dead staining indicated C17.2 cell survival on different proportions of composite hydrogels (G_5_H_0_+bFGF, G_5_H_0.25_+bFGF, G_5_H_0.5_+bFGF, G_5_H_1_+bFGF), DSC, and DSC+GH+bFGF, with dashed lines marking decellularized scaffold boundaries. (B) Bar chart showed that there is no significant difference in the 5-day survival rate of C17.2 cells on each scaffolds. (C) Live/dead staining showed NSCs distribution on composite hydrogels and DSC, with dashed lines indicating DSC boundaries. (D) EdU staining of C17.2 cells on different proportions of composite hydrogels (G_5_H_0_+bFGF, G_5_H_0.25_+bFGF, G_5_H_0.5_+bFGF, G_5_H_1_+bFGF) showed proliferating cells in green. (E) Bar chart showed that G_5_H_0.5_+bFGF exhibited the optimal cell proliferation among the different proportions of composite hydrogels. Scale bars = 100 µm in (A, C, D). Data are expressed as the mean ±SD; *P < 0.05, **P < 0.01, ***P < 0.001; *n* = 6.


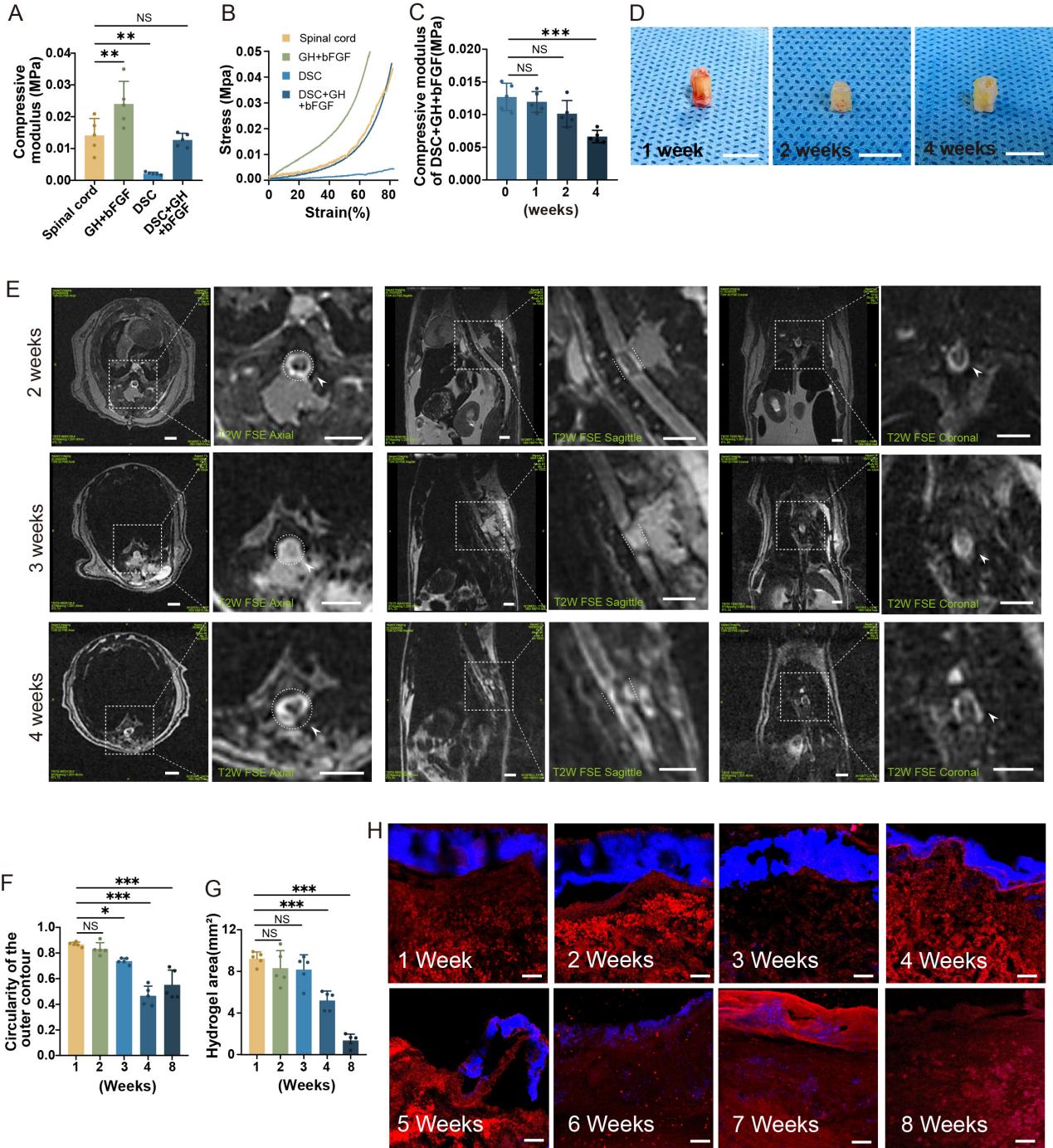


**Fig. S3: Evaluation of the mechanical properties, 3D structure and degradability of the lineage-specific matrix in vivo.** (A) Compressive modulus values showed that the rigid hydrogel shell improved the weak compression resistance of DSC, thereby achieving a compression modulus comparable to that of a fresh spinal cord. (B) Stress-strain curve showed the changes in fresh spinal cord, GH+bFGF hydrogel, DSC, and lineage-specific matrix under compressive force. (C) The compressive modulus of the lineage-specific matrix before implantation and at 1, 2, and 4 weeks after implantation. (D) Appearance of the lineage-specific matrix at 1, 2, and 4 weeks post-implantation. (E) Representative T2W MRI of the spinal cord implant area at 2, 3, and 4 weeks after the lineage-specific matrix was implantation. The right images correspond to enlarged images of selected areas in axial, sagittal and coronal views, respectively. (F-G) The outer contour circularity and cross-sectional area of the lineage-specific matrix during the first four weeks and at the 8th week after implantation. (H) The hydrogel shell gradually degraded 8 weeks after implantation of the lineage-specific matrix in the rat spinal cord. Scale bars = 5 mm in (D, E), 100 µm in (H). Data are expressed as the mean ±SD; *P < 0.05, **P < 0.01, ***P < 0.001; *n* = 5.


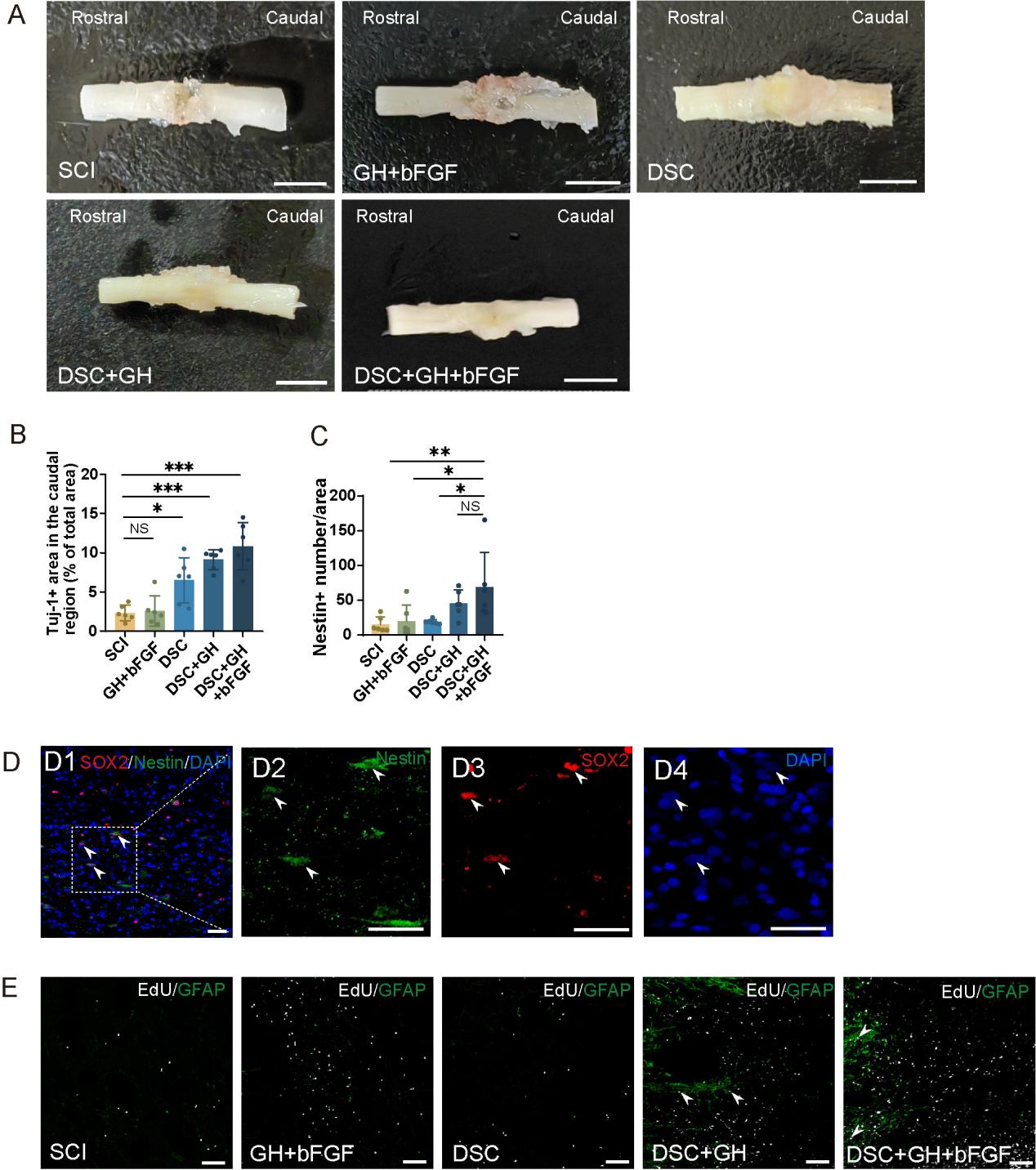


**Fig. S4: Appearance and immunofluorescence of the spinal cord post-injury/implantation.** (A) Appearance of the spinal cord in rats 8 weeks after SCI/implantation. (B) Bar charts showed the proportion of Tuj1+ area in the caudal injury/graft regions across five groups. (C) The bar chart showed the number of endogenous Nestin-positive cells in the injury/graft area of the five groups. (D) One week post-injury/implantation, SOX2 (red, arrowheads) and Nestin (green, arrowheads) double fluorescent staining showed that most endogenous NSCs in the injury/graft area co-express these markers. (E) Edu-labeled GFAP-positive astrocytes (green, arrowheads) in the injury/graft area of each group at 8 weeks post-injury/implantation, corresponding to the high-magnification area of Fig. 5H to L, respectively. Scale bars = 5 mm in (A), 100 µm in (E), 40 µm in (D). Data are expressed as the mean ±SD; *P < 0.05, **P < 0.01, ***P < 0.001; *n* = 6.


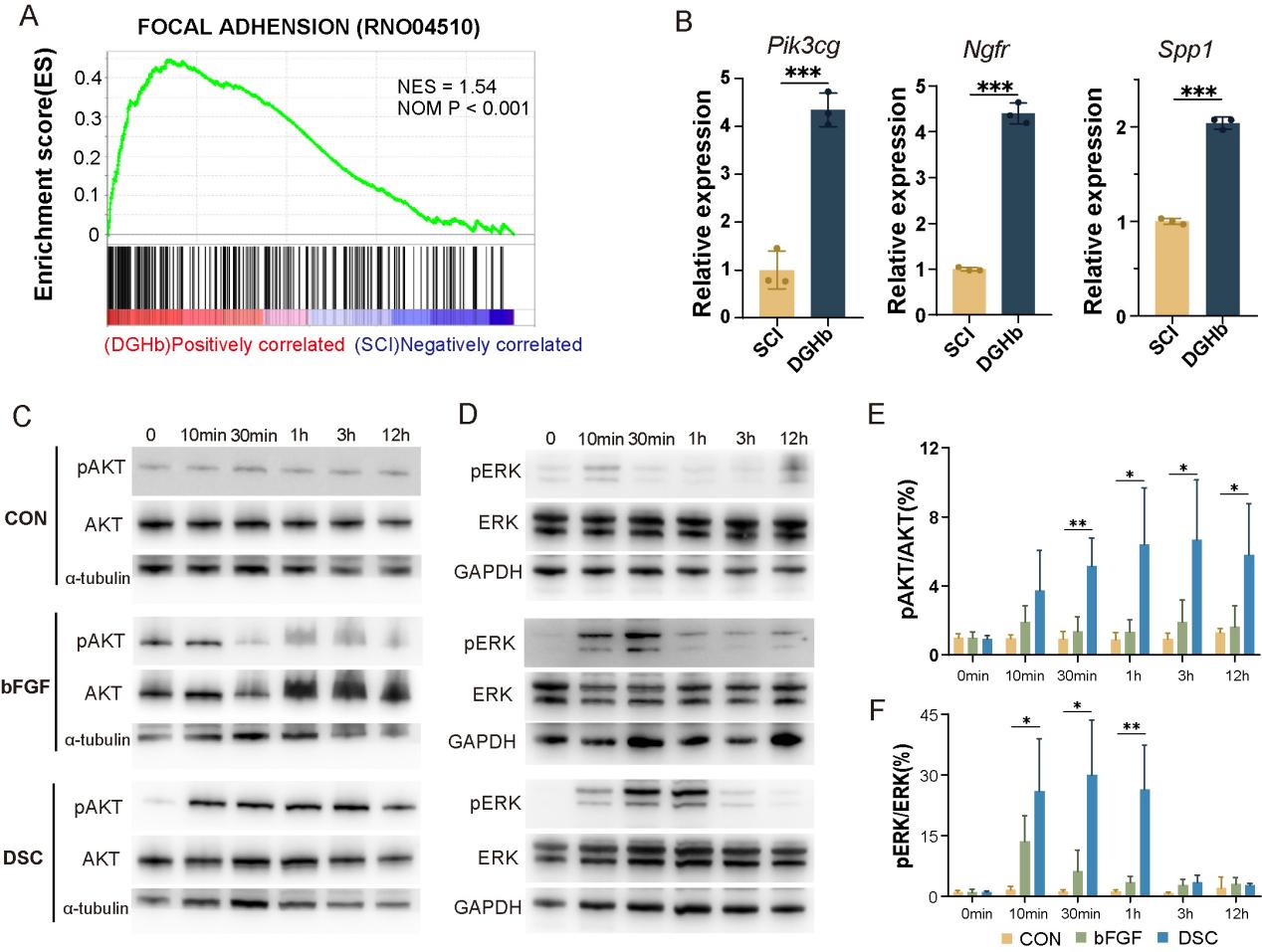


**Fig. S5: The DSC regulated NSCs through AKT/ERK phosphorylation.** (A) GSEA analysis revealed a significant enrichment of genes associated with focal adhesion in the injury/graft area following lineage-specific matrix implantation. (B) qRT-PCR analysis verified the results of the RNA-seq analysis. (C-D) Representative immunoblot images illustrated the phosphorylation levels of AKT and ERK in NSCs cultured under control conditions, with bFGF, and in DSC supplemented media at specified time points. (E-F) The intensity of each band was normalized to their respective α-tubulin or GAPDH loading controls, and the normalized p-AKT/AKT and p-ERK/ERK ratios are presented. Data are expressed as the mean ±SD; *P < 0.05, **P < 0.01, ***P < 0.001; *n* = 3.
