# Supplementary material for: Structure-Inspired Lineage-Specific Matrix for Endogenous Neurogenesis in Spinal Cord Injury
Source: Research (Wash D C). 2025 Aug 7;8:0821. doi: 10.34133/research.0821 (PMC12329214; doi:10.34133/research.0821)
Supplement: Supplementary 1 — Figs. S1 to S5 Tables S1 and S2 Movies S1 to S3 [file research.0821.f1.zip › supplementary tables-revised.docx]

**Supplementary Table 1**

Primary and secondary antibodies.

| Antibodies | Species | Catalog Number | Dilution | Source |
| --- | --- | --- | --- | --- |
| Laminin (LN) | Rabbit | L9393 | 1:100 | Sigma, St. Louis, USA |
| Chondroitin sulfate proteoglycans (CSPG) | Rabbit | BS-23788R | 1:100 | Bioss, Beijing, China |
| Microtubule associated protein 2 (Map2) | chicken | ab5392 | 1:500 | Abcam, London, UK |
| Glial fibrillary acidic protein (GFAP) | Rabbit | ab7260 | 1:2000 | Abcam, London, UK |
| Glial fibrillary acidic protein (GFAP) | Mouse | BM0055 | 1:100 | Boster Bio, Wuhan, China |
| CD68 | Rabbit | ab283654 | 1:50 | Abcam, London, UK |
| Iba1 | Goat | 011-27991 | 1:250 | FUJIFILM Wako Pure Chemical Corporation, Osaka, Japan |
| CD206 (Mannose receptor) | Rabbit | ab64693 | 1:200 | Abcam, London, UK |
| Tuj-1（β-Tubulin III） | Rabbit | ab18207 | 1:1000 | Abcam, London, UK |
| Nestin | Rabbit | ab92391 | 1:250 | Abcam, London, UK |
| Sox2 | Mouse | ab79351 | 1:200 | Abcam, London, UK |
| Goat Anti-Chicken IgY H&L (Alexa Fluor® 555) | Goat | ab150170 | 1:500 | Abcam, London, UK |
| Donkey anti-Rabbit IgG (H+L) Highly Cross-Adsorbed Secondary Antibody, Alexa Fluor™ 488 | Donkey | A21206 | 1:500 | Thermo Fisher Scientific, Waltham, USA |
| Donkey anti-Rabbit IgG (H+L) Highly Cross-Adsorbed Secondary Antibody, Alexa Fluor™ 555 | Donkey | A31572 | 1:500 | Thermo Fisher Scientific, Waltham, USA |
| Donkey Anti-Goat IgG H&L (Alexa Fluor® 488) | Donkey | ab150129 | 1:500 | Abcam, London, UK |
| Goat Anti-Mouse IgG H&L (Alexa Fluor® 488) | Goat | ab150113 | 1:500 | Abcam, London, UK |
| Goat Anti-Mouse IgG H&L (Alexa Fluor® 555) | Goat | ab150114 | 1:500 | Abcam, London, UK |
| Goat Anti-Rat IgG H&L (Alexa Fluor® 555) | Goat | ab150158 | 1:500 | Abcam, London, UK |
| AKT | Rabbit | C67E7 | 1:1000 | Cell Signaling Technology，Massachusetts, USA |
| Phospho-AKT | Rabbit | #4060 | 1:2000 | Cell Signaling Technology，Massachusetts, USA |
| ERK 1/2 | Rabbit | BM4326 | 1:500 | Boster Bio, Wuhan, China |
| Phospho-ERK1/2 | Rabbit | BM4156 | 1:500 | Boster Bio, Wuhan, China |
| GAPDH | Mouse | 60004-1-Ig | 1:50000 | Proteintech, Wuhan, China |
| ɑ-tubulin | Mouse | Clone 5-B-1-2 | 1:500 | Proteintech, Wuhan, China |
| HRP Conjugated AffiniPure Goat Anti-Rabbit IgG (H+L) | Goat | BA1054 | 1:10000 | Boster Bio, Wuhan, China |
| HRP Conjugated AffiniPure Goat Anti-Mouse IgG (H+L) | Goat | BA1050 | 1:10000 | Boster Bio, Wuhan, China |

**Supplementary Table 2**

Primers used for RT-qPCR.

|  | Forward | Reverse |
| --- | --- | --- |
| Pik3cg | 5′‐ATAGACCACCGCTTCCTCCT‐3′ | 5′‐CTGTTCCTCTGCCTTCCCAG‐3′ |
| Itga11 | 5′‐CCCCATCTGGATCATCGTGG‐3′ | 5′‐CTCTTGCGCTTGGCACTTTT‐3′ |
| Ngfr | 5′‐ATTCTCCGATGTGGTGAGCG‐3′ | 5′‐ATCTGCACACTGCATCGTCT‐3′ |
| Itga2 | 5′‐TGAGGTTTGGCATAGCGGTT‐3′ | 5′‐GTACCTCTCCGTTGGAGTGC‐3′ |
| Spp1 | 5′‐GATGACGACGACGATGACGA‐3′ | 5′‐GCTGGCAGTGAAGGACTCAT‐3′ |
| GAPDH | 5′‐GACATGCCGCCTGGAGAAAC‐3′ | 5′‐AGCCCAGGATGCCCTTTAGT‐3′ |
